# Mitochondrial Sirtuin 3: New emerging biological function and therapeutic target

**DOI:** 10.7150/thno.45922

**Published:** 2020-07-09

**Authors:** Jin Zhang, Honggang Xiang, Jie Liu, Yi Chen, Rong-Rong He, Bo Liu

**Affiliations:** 1 State Key Laboratory of Biotherapy and Cancer Center and Department of Gastrointestinal Surgery, West China Hospital, Sichuan University, and Collaborative Innovation Center of Biotherapy, Chengdu 610041, China.; 2 Anti-Stress and Health Research Center, College of Pharmacy, Jinan University, Guangzhou, Guangdong 510632, China.

**Keywords:** SIRT3, Mitochondrial homeostasis, Age-related disease, Cancer, SIRT3 activator, SIRT3 inhibitor

## Abstract

Sirtuin 3 (SIRT3) is one of the most prominent deacetylases that can regulate acetylation levels in mitochondria, which are essential for eukaryotic life and inextricably linked to the metabolism of multiple organs. Hitherto, SIRT3 has been substantiated to be involved in almost all aspects of mitochondrial metabolism and homeostasis, protecting mitochondria from a variety of damage. Accumulating evidence has recently documented that SIRT3 is associated with many types of human diseases, including age-related diseases, cancer, heart disease and metabolic diseases, indicating that SIRT3 can be a potential therapeutic target. Here we focus on summarizing the intricate mechanisms of SIRT3 in human diseases, and recent notable advances in the field of small-molecule activators or inhibitors targeting SIRT3 as well as their potential therapeutic applications for future drug discovery.

## Introduction

Sirtuins, a family of NAD^+^-dependent protein deacetylases, contain seven members (SIRT1-7) in mammals while bacteria and archaea possess only one or two members 1. Sirtuins are divided into five subclasses based on the conserved catalytic core domain. Class I is comprised of SIRT1, 2, and 3, which exhibit robust deacetylase activity. SIRT4, a class II Sirtuin, functions predominantly as an ADP-ribosyltransferase in mitochondria. SIRT5 belongs to Class III, while SIRT6 and 7 are assigned to Class IV. The U class sirtuins have only been observed in bacteria. Although subcellular localization and functions are different, mammalian sirtuins have different divisions of labor from regulating genome stability and energy metabolism to responding to cellular stress, working together to regulate the fate of cells as well as participating in various disease processes (Table 1) 2-4. Among them, SIRT3 localizes mainly to the mitochondrial matrix and plays an important role in regulating mitochondrial metabolism, including the tricarboxylic acid (TCA) cycle, the urea cycle, amino acid metabolism, fatty acid oxidation, ETC/oxidative phosphorylation (OXPHOS), ROS detoxification, mitochondrial dynamics and the mitochondrial unfolded protein response (UPR) 5, 6.

**Figure 1:**
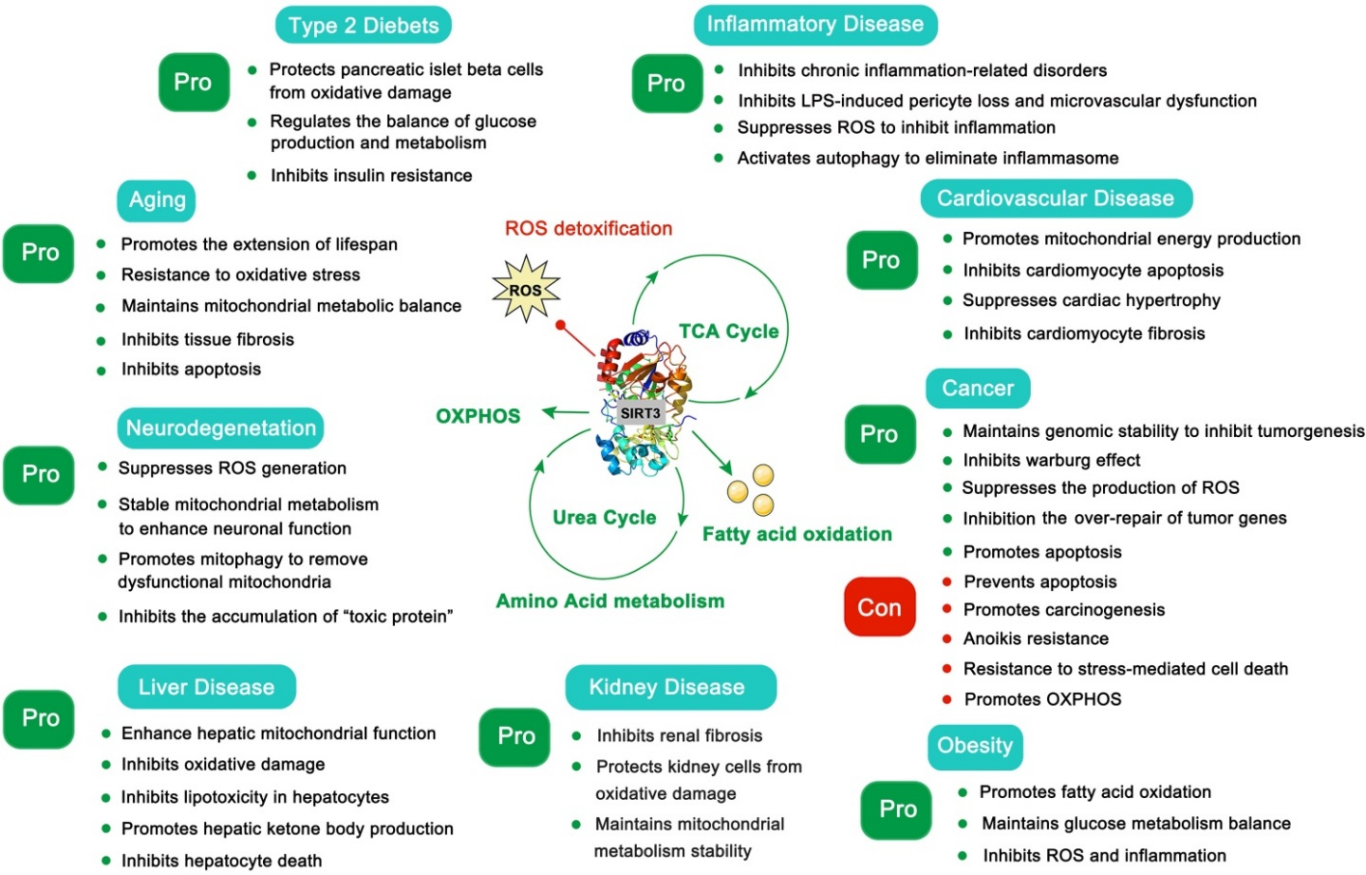
The pros and cons of SIRT3 in type 2 diabetes, aging, neurodegeneration, liver disease, inflammatory disease, cardiovascular disease, cancer, kidney disease and obesity.

The SIRT3 protein is widely expressed in mitochondria-rich tissues, such as kidney, heart, brain and liver tissue 12. The acetylation modifications that are regulated by SIRT3 are essential for maintaining mitochondrial function in these tissues. Given the crucial role of mitochondria in energy generation, metabolism, apoptosis and intracellular signaling 13, these highly metabolic tissues are more sensitive to mitochondrial dysfunction. Not surprisingly, SIRT3 has been verified to regulate aging, neurodegeneration, liver disease, kidney disease, heart disease and other metabolic diseases (Figure 1) 13. Moreover, the dual role SIRT3 plays in cancer development is intriguing 14. Therefore, SIRT3 has been proposed to be a promising therapeutic target for multiple human diseases, and a series of small-molecule compounds targeting SIRT3 have displayed favorable therapeutic effects 15, 16. In this review, we focus on summarizing the intricate mechanisms of SIRT3 in human diseases, the development of small molecule compounds targeting SIRT3 as well as their potential applications, and the potential superiorities and shortcomings regarding future drug discoveries with SIRT3 as a potential druggable target.

## Structure and function of SIRT3

**Figure 2:**
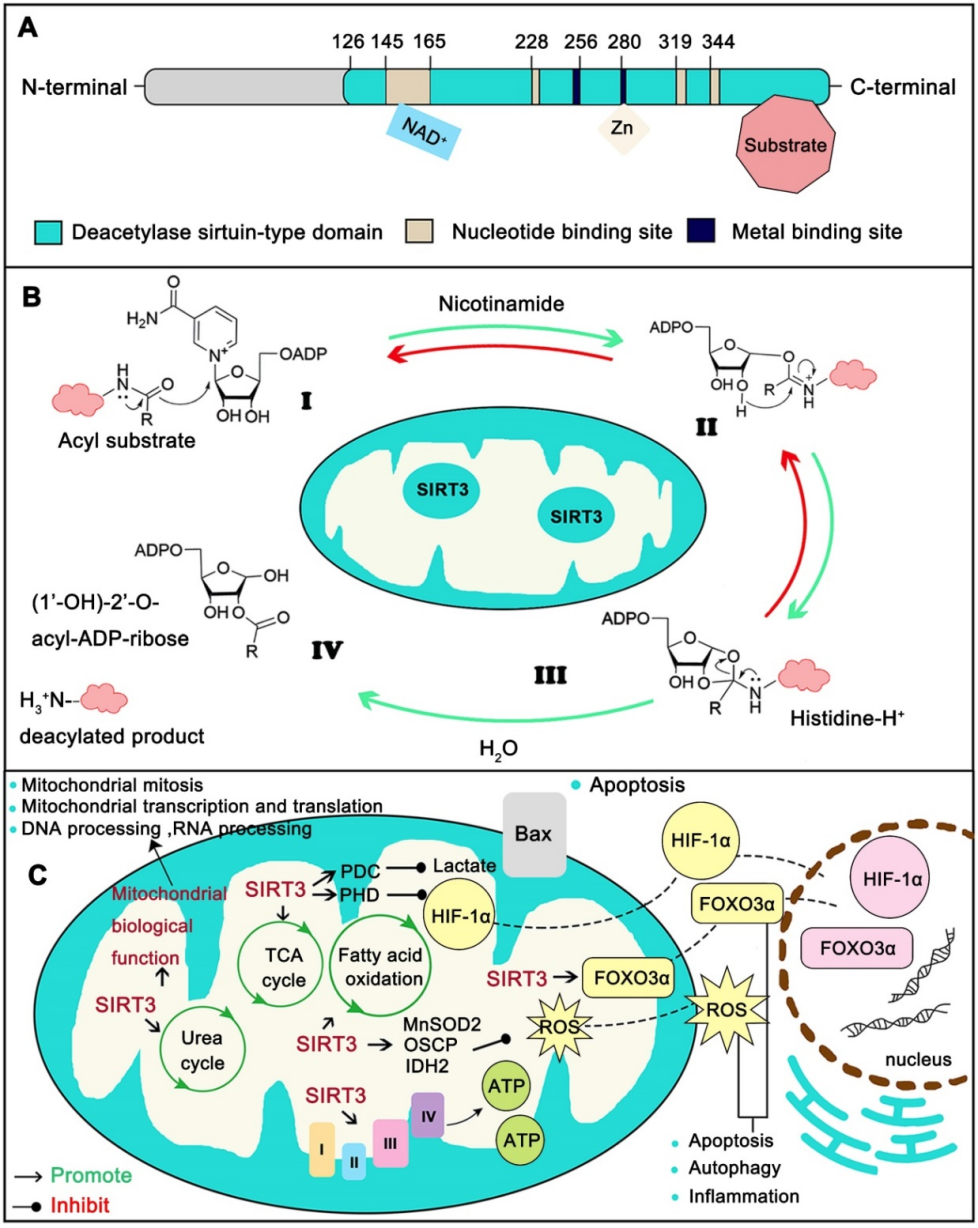
** Structure and function of SIRT3.** (**A**) The conserved enzymatic core of SIRT3 contains a NAD^+^ binding domain, a zinc binding motif and the binding sites of SIRT3 substrates. (**B**) The modification by SIRT3 is deacetylate its substrate with a NAD^+^ dependent manner. (**C**) Typical SIRT3 regulated biological function. SIRT3 assists mitochondria to maintain metabolic stability including the homeostasis of TCA cycle, Urea cycle, Amino acid metabolism, Fatty acid oxidation, ETC/OXPHOS, ROS detoxification and mitochondrial dynamics. Moreover, SIRT3 is closely related with oxidative stress, apoptosis, autophagy and inflammation.

As a typical sirtuin, SIRT3 has a conserved enzymatic core (aa126-399) undertaking the deacetylation function and acts in an NAD^+^ dependent manner. The core region contains a large Rossmann fold domain for NAD^+^ binding and a smaller domain comprising of a helical bundle and a zinc-binding motif which is formed by two loops extending from the large domain. The remainder of the enzymatic core is composed of the binding sites for SIRT3 substrates (Figure 2A) 14. Strictly speaking, the NAD^+^-dependent SIRT3 deacetylation reaction process includes four steps. First, the acetylated substrate and the NAD^+^ co-substrate bind in a cleft between the Rossmann-fold and zinc-binding domains, thereby inducing closure of the active site and stabilization of the NAD^+^ binding loop. Then the nicotinamide moiety of NAD^+^ is induced and buried in the nearby highly conserved hydrophobic pocket in a productive conformation. Subsequently, this conformation orients the α-face of NAD^+^ toward the acyl substrate, exposing the C1 atom of the ribose ring for direct nucleophilic attack from the carbonyl oxygen of acyl substrate. The acetyl group consequently transfers from the substrate to the ADP-ribose moiety of NAD^+^ coupled with cleavage of the nicotinamide from NAD^+^. Next, the C1'-*O*-alkylamidate intermediate converts to the bicyclic intermediate with the aid of the conserved His 224, which induces a nucleophilic attack of the 2'-OH group of the ribose onto the iminium carbon of the *O*-alkylamidate intermediate. Finally, the bicyclic intermediate is disrupted by an activated water molecule to produce the deacetylated protein and 2'-*O*-acetyl-ADP-ribose (Figure 2B). SIRT3 not only removes acetyl groups, but also crotonyl and myristoyl groups. However lysine acetylation is the most extensively studied modification 17. Mitochondrial proteins are relatively highly acetylated (nearly 20%) 18 and acetylation modification is involved in all aspects of mitochondria function. Therefore, acetylation modification is pivotal to mitochondrial destiny. In a recent report, the most important mitochondria sirtuin, SIRT3, can directly interact with at least 84 mitochondria proteins. These proteins are involved in all aspects of mitochondria biological function, including mitochondrial mitosis (CHCHD3, IMMT), transcription (TFAM, MTIF2, etc.), translation (MRPL11, MRPS34, etc.), DNA processing (POLB, POLDIP2,etc.), RNA processing (PNPT1, RNMTL1, etc.), lipid metabolism (ACADM, AGK , etc.), ETC/OXPHOS (NDUFA5, ATP5B and etc.), the TCA cycle (OGDH, DLST and etc.), as well as amino acid metabolism (GLUD1, OAT, etc.) 19. Together, SIRT3 supports the maintenance of mitochondrial homeostasis by regulating the acetylation levels of its substrates (Figure 2C).

## Endogenous regulators of SIRT3

SIRT3 is important in mitochondria, and therefore it is regulated in various ways. There are several studies on endogenous regulators of its expression, as well as transcriptional and post-translational modifications on this protein (Table 2). SIRT3 is a sensor of mitochondrial energy, thus levels of the metabolic co-factor (NAD^+^) and the byproduct nicotinamide are direct regulators of its activity. As the co-factor of SIRT3, NAD^+^ promotes the deacetylation process 20 while nicotinamide inhibits this process through accelerating the reverse reaction by binding to the reaction product 17, 21. In addition, caloric restriction (CR) is another crucial factor that can obviously stimulate the expression of SIRT3, which is also a biological self-protection strategy. In response to CR, the up-regulated SIRT3 activates mitochondrial isocitrate dehydrogenase 2 (IDH2) by deacetylation, thus increasing the NADPH level to reduce oxidative damage and enhance the mitochondrial antioxidant defense system 20. SIRT3 is transcribed in the nucleus and full-length SIRT3 (FLSIRT3) has no enzyme activity. FLSIRT3 is imported into the mitochondria, then its NH_2_ terminus is proteolytically cleaved in the mitochondrial matrix to become the active form, SIRT3 (an approximately 28kDa product). During this process, matrix processing peptidase (MPP) acts as “scissors” to accomplish this post-translational modification 22. SUMOylation is another post-translational modification on SIRT3, which further inhibits its activity. SUMO specific protease SENP1 can de-SUMOylate and activate SIRT3 to promote mitochondria metabolism 23. SIRT3 can also be regulated by covalent modification. 4-Hydroxynonenal (4-HNE) is an endogenous product of lipid peroxidation which inhibits SIRT3 activity by occupying its zinc-binding residue (Cys 280) 24. In addition, transcriptional modification is another major regulation of SIRT3. NF-κB, which is a pleiotropic transcription factor, was recently identified to bind to the SIRT3 promoter to enhance its expression 25. Peroxisome proliferator-activated receptor γ (PPARγ) coactivator 1 (PGC-1α) could bind to the SIRT3 promoter to stimulate SIRT3 transcription 26, 27, while SNAI1 and Zinc finger E-box-binding homeobox 1 (ZEB1) negatively regulates the SIRT3 promoter activity to inhibit its expression 28, 29. Of note, microRNAs (miRNAs) are mainly post-transcriptional regulators that affect mRNA stability and protein levels which can also regulate SIRT3 activity. As a class of non-coding RNA molecules, miRNAs bind to complementary target mRNAs, resulting in mRNA translational inhibition or degradation 30. Studies have proven that miR-195 31, miR-421 32, miR-494-3p 33, miR-708-5p 34, miR-31 35, miR-145 36, miR-298 37 could directly target the 3'UTR of SIRT3, thus inhibiting its gene expression and protein levels. In addition, miR-210 targets and represses the iron-sulfur cluster assembly protein (ISCU), which changes the NAD^+^/NADH ratio, indirectly influencing SIRT3 38. Long non-coding RNA (LncRNAs) play a similar role to miRNAs as well. LncRNA TUG1 suppresses the mRNA expression of miR-145, which can further positively regulate SIRT3 36, while LncRNA DYNLRB2-2 inhibits miR-298 to activate the transcription of SIRT3 37. The protein-protein network is another primary method of regulating SIRT3 activity. Profilin1, an actin-associated protein, can interact with SIRT3 and promote its expression 39. β-catenin, a key downstream effector in the Wnt signaling pathway, can suppress SIRT3 promoter activity thus inhibiting its expression 40. As mentioned above, these endogenous regulators modulate the expression and activities of SIRT3 in response to cellular stresses, thus maintaining homeostasis.

## SIRT3 and human disease

### “Small” modification, “big” function

The biological function of SIRT3, which is just deacetylation of its substrate, seems to be relatively simple and limited, but it does participate in numerous biological processes and the development of various diseases 41, 42. The most fascinating role of SIRT3 is in longevity. It was first reported in 2003 that SIRT3 is positively associated with longevity 43, and much subsequent work has confirmed and expanded this result 44-46. More delightfully, studies have proven that SIRT3 protects human heart, brain, muscle, liver, kidney and other tissues from dysfunction and disease 46. SIRT3 is also involved in the overall progression of cancer. It may function differently in various conditions, but it mostly inhibits the development of cancer 14. Here, we will discuss how SIRT3 fights against human diseases by regulating specific substrate proteins and their corresponding signaling pathways.

### SIRT3 in age-related disease

**Figure 3:**
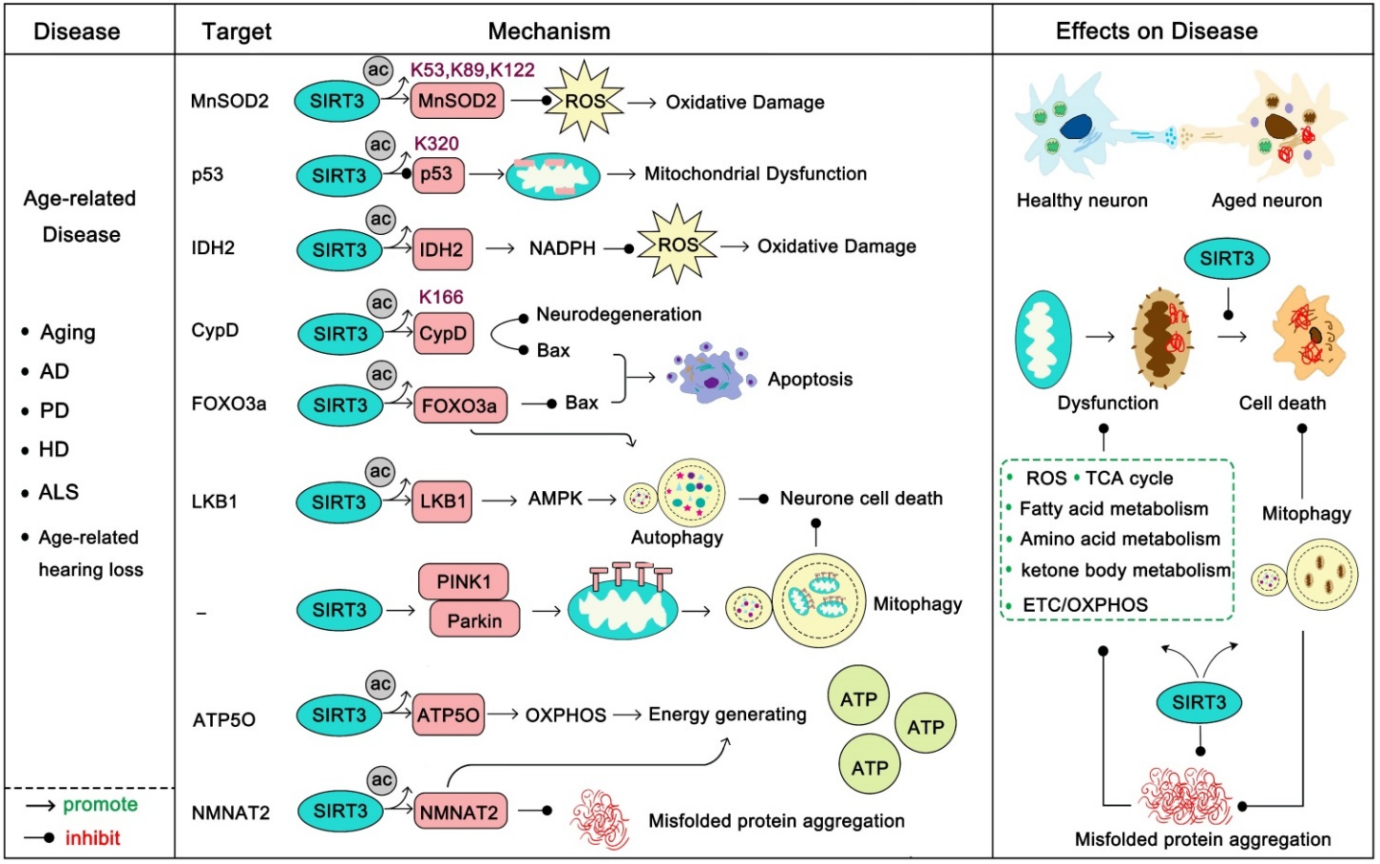
** SIRT3 in age-related disease.** Age-related diseases are always accompanied with a decline in mitochondrial function, high oxidative stress and accumulation of toxic proteins. SIRT3 activates a range of substrates by deacetylation to promote mitochondrial function, enhance ATP production, accelerate ROS clearance, and maintain mitochondrial metabolic homeostasis. In addition, SIRT3 can activate mitophagy to accelerate mitochondrial renewal. It is worth noting that SIRT3 also inhibits the production of misfolded proteins and accelerates their clearance. The pink proteins represent the substrates of SIRT3. Gray circles represent acetylation modifications.

Age-related disease usually occurs with high frequency after a certain point, and these diseases are always accompanied by the gradual decline of function in various organs. In this section, we mainly focus on aging, Alzheimer's disease (AD) 47, Parkinson's disease (PD) 48, Huntington's disease (HD) 49, amyotrophic lateral sclerosis (ALS) 50 and age-related hearing loss (Figure 3). Aging is a complex and irreversible process. Although the pathological features of aging vary considerably, they all bear the common characteristic that the smallest components of lesions, such as neuronal or muscle cells, have decreased or imbalanced mitochondrial function and increased oxidative damage 51. Under normal circumstances, SIRT3 is highly expressed in the brain and nervous system, but decreased SIRT3 expression is always observed in age-related diseases and this age-dependent defect of SIRT3 is a major risk factor of pathogenesis 52-54.

Another well-studied role of SIRT3 in age-related disease is that it can act to resist apoptosis. Progressive loss and death of neuronal cells is a feature of these diseases, and apoptosis plays an important role in this process.* P53*, the most famous tumor suppressor gene, is highly acetylated in neurodegenerative diseases. Ac-p53^K320^ targets mitochondria and directly reduces its function, inducing mitochondrial dysfunction which leads to mitochondrial-dependent apoptosis. SIRT3 deacetylates p53 to prevent its localization-induced releasing of cytochrome c from the mitochondria to the cytoplasm 53. Moreover, SIRT3 activates FOXO3α, a transcription factor of the FOXO family which is involved in metabolism-regulation and stress response, to inhibit Bax-regulated apoptosis 57. SIRT3 can deacetylate CypD, a regulatory component of the mitochondrial permeability transition pore (mPTP), to inhibit the mitochondrial permeability transition to prevent apoptosis and mitochondrial dysfunction 58. In response to age-dependent mitochondrial capacity reduction, SIRT3 binds to ATP5O, a mitochondrial ATP synthase, strengthening the ETC/OXPHOS process to enhance cellular ATP production 19. Recently, studies have shown that the activation of SIRT3 and the inhibition of apoptosis play an important role in the improvement of age-related diseases. For example, celastrol can ameliorate age-related macular degeneration via SIRT3 activation and apoptosis inhibition 59. Melatonin decelerated age-induced ovarian aging via activating SIRT3-regulated mitochondrial function enhancement together with apoptosis inhibition 60. Not surprisingly, daily melatonin supplementation protects vascular endothelium from aging via SIRT3-regulated oxidative stress suppression and apoptosis inhibition 61. Accordingly, up-regulation of SIRT3 plus apoptosis inhibitors might provide new directions for age-related disease treatment. SIRT3 can activate autophagy, a notable cellular self-protective mechanism. SIRT3 can deacetylate LKB1 to activate the AMPK-mTOR autophagy pathway 62. More interesting, deacetylated FOXO3α can activate expression of various autophagy genes (*ULK1*,* ATG5*,* ATG7*, et al) to protect cells from apoptosis 63. Mitophagy is indispensable for maintaining mitochondrial homeostasis and is a specific form of autophagy that selectively removes dysfunctional mitochondria. When an organ is unable to repair dysfunctional mitochondria, mitophagy helps to provide for metabolic needs and contributes to mitochondrial renewal 64. SIRT3 induces the initiation and activation of the PINK1-Parkin mitophagy pathway to inhibit cell death 65. Induction of autophagy is a new hotspot in the treatment of neurodegenerative diseases 66, 67. Therefore, SIRT3 activation along with autophagy activators (like ULK1 activators 68, 69, rapamycin 70) will be a new candidate method for treatment of these diseases. Of note, another major pathogenesis of age-related disease is the aggregation of pathological proteins (such as Aβ, tau in AD, and α-synuclein in PD) 47-50. Recently, nicotinamide mononucleotide adenylyltransferase 2 (NMNAT2), a substrate of SIRT3, was found to be a neuron protector by interacting with heat shock protein 90 (HSP90) to refold aggregated protein substrates 71. SIRT3 can interact with NMNAT2 and activate it to improve mitochondrial function and inhibit apoptosis 72. More interestingly, NMNAT2 is a neuron-specific and effective protector which inhibits axonal degeneration and dysfunction, and it is very likely that SIRT3 can promote this function 73. It is noteworthy that SIRT3-induced autophagy might also be an effective method to eliminate these pathological proteins, thus SIRT3 activation together with “toxic protein” scavenging drugs might be beneficial. Taken together, SIRT3 is the nemesis of age-related diseases and it fights against almost all the aspects of their development. Properly targeting SIRT3 will be promising in these diseases.

### SIRT3 in cancer

Cancer cells exhibit metabolic patterns that are distinct from normal cells. The feature of mitochondria metabolic reprogramming is the “Warburg effect” 74, which is that most cancer cells are more dependent on aerobic glycolysis than oxidative phosphorylation, regardless of the fact that glycolysis produces ATP inefficiently. Glycolysis not only provides a rapid energy supply, but also provides a number of favorable factors for the occurrence and development of tumor microenvironments, such as accelerating the instability of genome and the positive response to multiple cell proliferation signals (PI3K/AKT, c-Myc, etc.) 75, 76. Of course, in some specific cancers or specific circumstances (such as brain cancer and acute myeloid leukemia), OXPHOS is still their more suitable means of energy supply, and the survival of these cancers is more dependent on the continued activity of OXPHOS 77-79. The tumor microenvironment of different cancers varies considerably, and the pressure of survival makes them evolve to choose the most suitable metabolic pathway. In short, SIRT3 promotes oxidative phosphorylation and inhibits glycolysis in the overall regulation of mitochondrial metabolism 80, 81. Intriguingly, a recent study found that SIRT3 changes to an oncogene to promote HFD-induced tumorigenesis in mice 82. Therefore, the role of SIRT3 in cancer appears context-dependent 83. In most cases, it is carcinogenic in some cancers that are addicted to OXPHOS 77. However, it is also a tumor suppressor in glycolysis-dependent cancers 80, 81, 84. Since the role of SIRT3 in cancer has been widely reported and is well summarized, in this part we mainly focus on how SIRT3 regulates its substrates to play a double-sided role in cancer.

#### Tumor suppressor role of SIRT3

The most important tumor suppression role of SIRT3 is that it hinders cancer metabolism changes. The inhibition of hypoxia-inducible factor-1a (HIF1α) by SIRT3 is the most studied pathway. HIF1α is a key factor that activates a series of glycolysis genes which contribute to the “Warburg phenotype”. SIRT3 can destabilize HIF1α to prevent its deleterious role of “Warburg effect” promotion. Interestingly, HIF1α is not the direct substrate of SIRT3. SIRT3 regulates the activity of HIF1α by direct deacetylation of prolyl hydroxylase (PHD). Activated PHDs then hydroxylate HIF1α to affect its stability and decrease its tumor promoting effect 81. The pyruvate dehydrogenase complex (PDCs) is another SIRT3 substrate related to glycolysis. In the process of hypoxia and tumor growth, lysine acetylation of PDC plays an important role in promoting glycolysis and subsequent cancer cell proliferation. SIRT3 is the upstream deacetylase of PDC that deacetylates and activates PDC to inhibit glycolysis and promote apoptosis in cancer cells 85. In addition, SIRT3 could stabilize p53 to inhibit glycolysis in wt-p53 cancer cells. During this process, full-length SIRT3 can interact with *PTEN* in the nucleus to increase* PTEN* activity thus inhibiting MDM2 transcription, which is responsible for p53 degradation 86. The stabilization of p53 further impedes glycolysis via inhibition of the transcription of numerous key glycolytic enzymes 87. Moreover, SIRT3 can inhibit breast carcinoma glycolysis through deacetylation and inactivation of cyclophilin D. This further inhibits the binding of the lactate metabolism enzyme hexokinase II (HK II) to mitochondria to obstruct glycolysis 88. Glutamate oxaloacetate transaminase 2 (GOT2), which is a limiting enzyme in regulating glycolysis processing, is deacetylated at Lys 159, 185, and 404 by SIRT3 thus inhibiting GOT2 activity and hindering pancreatic tumor growth 89. Accordingly, in most glycolysis-addiction cancers, SIRT3 activation will be beneficial and SIRT3 activators might be candidate adjuvants. In addition, SIRT3 directly or indirectly inhibits ROS-regulated tumorigenesis and metastasis 90. Of which, SIRT3 represses ROS dependent Src/FAK signaling and promotes the proliferation, migration and metastasis of cancer cells 91. ROS elimination by SIRT3 was also proven to contribute to the growth inhibition of lung adenocarcinoma cells 92. In chronic lymphocytic leukemia (CLL), SIRT3 activates MnSOD2 to eliminate ROS, thus inhibiting CLL progression 93. Additionally, programmed cell death induction is another strategy SIRT3 uses to inhibit tumor progression. For instance, SIRT3 can activate glycogen synthase kinase-3β (GSK-3β) to promote the Bax-regulated apoptosis pathway 94. SIRT3 up-regulation of MnSOD2 and p53 activity further induced Bax- and Fas-regulated apoptosis in HCC 95. Moreover, SIRT3 plays a role in monitoring the stability of tumor genomes. Histone H3, which participates in the reaction to DNA damage, can be deacetylated by SIRT3 at K56 to enhance DNA nonhomologous end joining repair 96. 8-oxoguanine-DNA glycosylase 1 (OGG1) is a DNA repair enzyme that is important in the inhibition of genome damage. SIRT3 binds and blocks the degradation of OGG1 to inhibit tumorigenesis 97.

Of note, SIRT3 can also inhibit cancer progression by deacetylation of its substrate to modulate proliferation and migration. The F-box protein S-phase kinase associated protein 2 (Skp2) is a proto-oncogene which inhibits the activity of numerous tumor suppressor proteins (such as p21, p27 and E-cadherin) via ubiquitination and destruction to positively regulate cell cycle progression and migration, and negatively regulate apoptosis 98, 99. Acetylation of Skp2 by histone acetyltransferase p300 at Lys 68 and 71 will increase its stability and cytoplasmic localization, which promotes the degradation of E-cadherin. SIRT3 can directly deacetylate Skp2 to prevent this process 100. Similarly, Enoyl-CoA hydratase-1 (ECHS1) is also highly acetylated in cancer cells. Acetylation blocks the activity of ECHS1 and activates the mTOR-regulated proliferation pathway. SIRT3 inhibits this hyperacetylation to restore mitochondrial translocation and ECHS1 activity 101. Hyperacetylation of Glutamate oxaloacetate transaminases (GOT) promotes tumor growth while SIRT3 reverses this process 89. It has been shown in recent studies that in some highly malignant tumors, the activation of SIRT3 might be a possible treatment method, especially for some drug-resistant cancers. For example, sorafenib is a well-known drug approved for clinical use in hepatocellular carcinoma (HCC), but is very prone to drug resistance, which makes its treatment of liver cancer less satisfactory. Interestingly, studies revealed that sorafenib could decrease the expression of SIRT3, which contributes to its reduced drug sensitivity, while up-regulation of SIRT3 can re-sensitize HCC to sorafenib treatment 102-104. In addition, SIRT3 activation by ABT737 contributes to ameliorating cisplatin resistance in ovarian cancer 105.

#### Oncogenic role of SIRT3

The tumor promoting effect of SIRT3 has also been well studied, especially in some hematological malignancies that exhibit oxidative phosphorylation addiction 77, 106. IDH2 is a key enzyme acting in the forward Krebs cycle and was identified as a hallmark of hematological malignancies. SIRT3 can deacetylate IDH2 to increase its activity in promoting carcinogenesis 107. In diffuse large B cell lymphomas (DLBCLs), SIRT3 was proven to accelerate TCA cycle metabolism via enhancing GDH activity to promote lymphomagenesis 108. Not surprisingly, SIRT3 has also been found to promote tumor progression in some other cancers. In non-small cell lung cancer, SIRT3 promotes the oncogenic role of NMNAT2 to stimulate cancer cell proliferation 72. Similarly, SIRT3 deacetylates p53 at Lys 320 and 382 to promote its degradation, thus hindering its tumor inhibition role in *PTEN*-deficient non-small cell lung cancer 109. Therefore, in *PTEN*-deficient tumors, SIRT3 inhibition might be a better treatment strategy. Recently, another study demonstrated that SIRT3 could promote colorectal carcinogenesis. SIRT3 deacetylates serine hydroxymethyltransferase 2 (SHMT2) at Lys 95 and inhibits its lysosome-dependent degradation. Acetylated SHMT2 exhibits deficient enzymatic activity, which inhibits carcinogenesis, while SIRT3 activates SHMT2 to promote colorectal cancer cell proliferation 39. In addition, SIRT3 can increase SOD2 activity to properly regulate ROS production to prevent apoptosis 110. In ovarian cancer cells, SIRT3 fine-tunes SOD2 activity to adapt to cellular stress and anoikis resistance in order to ensure cell survival 111. Interestingly, the activation of SOD2 mediated by SIRT3 can promote epithelial-mesenchymal transition (EMT) in triple negative breast cancer (TNBC) cells 25. In cervical cancer cells, SIRT3 deacetylates Acetyl-CoA carboxylase (ACC1) to promote lipid metabolism. This fatty acid metabolism reprogramming promotes cancer migration and invasion 112. Pyrroline-5-carboxylate reductase 1 (PYCR1) is another substrate of SIRT3. Acetylation at Lys 228 suppresses tumor proliferation, while deacetylation by SIRT3 promotes breast cancer cell and lung cancer cell survival 113. Accordingly, we should be thoughtful in trying to regulate SIRT3 for cancer therapy.

**Figure 4:**
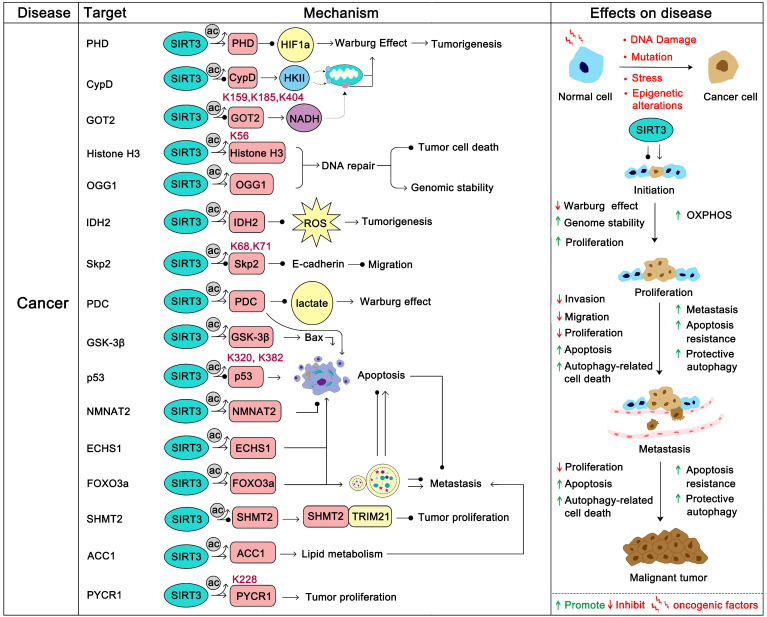
** SIRT3 in cancer.** SIRT3 plays a two-sided role in cancer. In most cancers, SIRT3 plays a tumor suppressor role. On the one hand, SIRT3 can maintain the stability of the cancer genome and inhibit carcinogenesis. On the other hand, SIRT3 inhibits the Warburg effect of cancer to inhibit the development of tumors. In addition, SIRT3 can inhibit tumor proliferation and metastasis. SIRT3 induced apoptosis and autophagy also involved in this progress. However, in some colorectal cancers and lung cancers, SIRT3 plays an oncogenic role by promoting proliferation and metastasis via deacetylation of specific substrates. The pink protein represents the substrate for SIRT3. Gray circles represent acetylation modifications.

Overall, SIRT3 is capable of metabolic reprogramming and contributes greatly in the fate of cancers (Figure 4). Highly acetylated modifications frequently occur in cancer, which is conducive to the survival of most tumors. SIRT3 regulates tumor progression by changing this excessive modification back to a normal condition. The role of SIRT3 in cancer is a double-edged sword which to some extent increases the confusion and risk of SIRT3 as a target for cancer treatment. More regrettably, there are no satisfactory SIRT3 activators or inhibitors that have been developed successfully for cancer therapy, which makes recognition of SIRT3 as a druggable target in cancer more difficult and questionable. Even so, we believe it is a promising drug target in cancer if we can intelligently regulate it in personalized therapy.

### SIRT3 in heart disease

**Figure 5:**
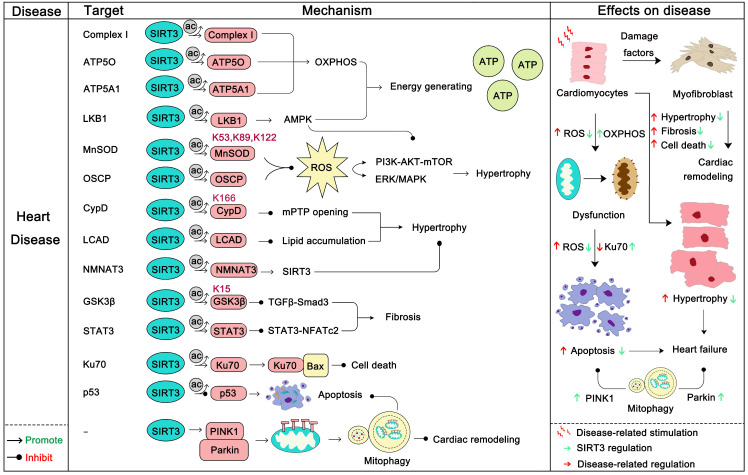
** SIRT3 in heart disease.** Heart disease often manifests as dysfunction of cardiomyocytes, such as local hypoxia, death of cardiomyocytes, fibrosis, and the like. Dysfunction of cardiomyocytes ultimately leads to myocardial ischemia, cardiac hypertrophy, heart failure. SIRT3 can increase the mitochondrial function of cardiomyocytes and increase energy production by deacetylating its substrate. In addition, SIRT3 can deacetylate its substrates to inhibit AKT-mTOR/ERK1/2/TGF-β-smad3-induced myocardial fibrosis. During this process, SIRT3 can also activate GSK-3β to contribute to myocardial fibrosis inhibition. SIRT3 also directly inhibits cardiomyocytes apoptosis. Last but not the least, SIRT3 can eliminate ROS and activate mitophagy to inhibit cardiac remodeling. The pink protein represents the substrate for SIRT3. Gray circles represent acetylation modifications.

Heart disease is one of the most common diseases and one of the biggest killers in the field of human health 97. The role of the heart is to encourage blood flow, provide sufficient blood to organs and tissues to supply oxygen and various nutrients, and remove metabolic products to maintain metabolism and homeostasis. The normal work of the heart requires huge amounts of energy production and consumption, which is mainly provided by the mitochondria 114. The normal function of mitochondria as energy-producing engines is the basis of the normal function of the heart. Conversely, the dysfunction of mitochondria directly or indirectly contributes to the progression of a series of heart diseases, among which are heart failure, cardiac hypertrophy, atherosclerosis, and dilated cardiomyopathy 115, 116. As an eminent guardian of mitochondrial homeostasis, SIRT3 also plays an irreplaceable role in heart disease. Loss of SIRT3 impairs the contractile function of the heart 117, and in a later study SIRT3 was found to be down-regulated in heart failure. In this process, miR-195 targets SIRT3 to inhibit its expression thus disturbing oxidative phosphorylation, resulting in the myocardial energy metabolism imbalance 31. Another study reported that rarefaction of cardiac microvessels and functional hypoxia are more likely to occur in SIRT3-KO mice than WT mice. And SIRT3-KO mice exhibit distinct mitochondrial dysfunction and enhanced cardiac fibrosis marker expression 118. Moreover, SIRT3-KO mice exhibited diastolic dysfunction and decreased angiogenesis 119. Remarkably, SIRT3 deletion mice are more susceptible to a high-fat diet and exhibit higher ROS production and lower cardiac function 120. In addition, SIRT3 can inhibit arterial thrombosis by suppressing neutrophil extracellular traps and decreasing plasma tissue factor activity 121. More interestingly, a study found that acetylation of mitochondrial proteins mostly occurs nonenzymatically and SIRT3 just modifies the acetylation back to the normal level. A more prominent role of SIRT3 is to remove acetylation lesions on its substrates, such as at Lys 127 on Glycine N-acyltransferase (GLYAT) and at Lys 48 on hydroxymethylglutaryl-CoA lyase (HMGCL), to protect metabolic fidelity 122. In this section we will mainly discuss how SIRT3 regulates its substrates to carry out its heart protector role (Figure 5).

The first protective effect of SIRT3 on the heart is to increase the energy production of mitochondria. SIRT3 can activate mitochondrial complex I and enhance the ETC function to maintain ATP homeostasis 123. ATP5O and ATP5A1, two mitochondrial ATP synthases, can be deacetylated by SIRT3 to enhance ATP production 19, 124. In addition, SIRT3 actives the LKB1-AMPK pathway to generate ATP 125. Optic atrophy 1 (OPA1), another target of SIRT3, can be triggered by SIRT3 to improve cardiac mitochondrial bioenergetics 126. In addition, SIRT3 can regulate a series of substrates to block cardiac hypertrophy. Cardiac hypertrophy is a stress adaptive response to a range of heart conditions, but it also increases mortality and the risk of heart disease. Thus, inhibiting hypertrophy may be beneficial to the heart. Of note, Rho/Rho kinase signaling, mTOR signaling, ROS signaling are three key villains of cardiac hypertrophy 127. Of note, the LKB1-AMPK pathway is the upstream negative regulator of mTOR signaling, and SIRT3 can active LKB1-AMPK signaling to inhibit mTOR regulated protein synthesis, which inhibits cardiac hypertrophy 125. More interestingly, the SIRT3 substrate FOXO3α not only functions well in regulating apoptosis and autophagy, but also it regulates mTOR and Rho/Rho kinase signaling 128, 129. SIRT3 can active the activity and nuclear translocation of FOXO3α, thereby inhibiting mTOR and Rho/Rho kinase signaling and preventing cardiac hypertrophy. To inhibit ROS regulated signaling, SIRT3 can directly deacetylate, activate MnSOD2 and oligomycin-sensitivity conferring protein (OSCP), inhibiting the synthesis and aggregation of ROS, thus preventing cardiac hypertrophy 16. Cyclophilin D (CypD), which is an integral part of the mitochondrial permeability transition pore (mPTP), can be deacetylated by SIRT3 at lysine 166 to prevent the opening of mPTP, thus inhibiting stress-induced cardiac hypertrophy and apoptosis 58. Hypertrophy-related lipid accumulation is another problem of cardiac hypertrophy, which will further lead to heart failure. SIRT3 could downregulate the acetylation of long-chain acyl CoA dehydrogenase (LCAD) to restore lipid metabolism homeostasis 130. In addition, Nicotinamide mononucleotide adenylyltransferase 3 (NMNAT3), can also bind to SIRT3 and be deacetylated. Deacetylation of NMNAT3 enhances its enzyme activity and in-turn promotes the anti-hypertrophic effects of SIRT3 131.

Cardiac fibrosis is another pathological manifestation of cardiac remodeling in heart disease. SIRT3 can ameliorate cardiac fibrosis through blocking the TGF-β/Smad3 pathway 132. SIRT3 can deacetylate glycogen synthase kinase 3β (GSK3β) at residue Lys 15 to promote GSK3β activity. The activation of GSK3β thereby resists TGF-β/Smad3 regulated fibrotic genes expression 133. Moreover, signal transducer and activator of transcription 3 (STAT3) can be deacetylated by SIRT3 to inhibit STAT3-NFATc2 regulated fibrosis 134. Also the role of SIRT3 in eliminating ROS also helps the SIRT3/ROS/ERK1/2 cascade inhibit cardiac remodeling 135. In addition, the role of SIRT3-induced autophagy and apoptosis inhibition in heart disease should not be ignored. Parkin-dependent mitosis induced by SIRT3 can clear damaged mitochondria and prevent the remodeling of hypertensive heart and the death of myocardial cells 65. Ku70, another substrate of SIRT3, can be deacetylated and activated by SIRT3. After deacetylation, Ku70 interacts with Bax to inhibit the apoptosis of cardiomyocytes 136. SIRT3 can protect cardiomyocytes by inhibiting apoptosis through deacetylating p53. As mentioned above, SIRT3 has a wide protective role in heart disease, and targeting SIRT3 can be a new strategy for the treatment of heart disease. It is gratifying that the SIRT3 activator honokiol has been proven to have cardioprotective effects 16, 137, which also provides evidence for SIRT3 as a druggable target for improving heart disease.

### SIRT3 in metabolic disease

Metabolic diseases generally have a long course over which energy metabolism, glycometabolism, fatty acid metabolism, and amino acid metabolism gradually become out of balance. Eventually this leads to obesity, diabetes, liver disease, and kidney disease 138. The kidney is a highly energy-consuming organ because it not only removes metabolites from the body by generating urine, but also maintains the balance between water and electrolytes. Additionally, it secretes some active substances, such as erythropoietin and active vitamin D3 to promote the growth and development of the body 139. The kidney is rich in mitochondria in order to satisfy its energy needs, and the mitochondrion is a highly mobile organelle that can change its position and numbers as needed 140. SIRT3, as a key regulator of mitochondrial dynamics, plays an important role in the energy supply of renal cells 141. The overexpression of SIRT3 can improve kidney function, attenuate oxidative injury, and suppress the inflammatory damage and apoptosis of renal tubular epithelial cells 142. The absence of SIRT3 expression will aggravate acute renal injury (AKI), and increase ROS levels and apoptosis. Activation of SIRT3 decreases the acetylation of CypD, thereby inhibiting mitochondrial damage of AKI, and thus protecting the kidneys 143, 144. Activation of SIRT3 also inhibits the acetylation of p53 which blocks apoptosis in AKI 145. In addition, SIRT3 deacetylates PGC1-α and mitochondrial complex I to enhance mitochondrial biogenesis and energy generating for resisting AKI 139. Nephrolithiasis is a form of kidney metabolic disease and its main cause is damage to renal epithelial cells from calcium oxalate. A recent study found that nephrolithiasis-afflicted mice always exhibit a significant reduction in SIRT3 expression. SIRT3 could deacetylate FOXO3a thus activating it. After deacetylation, FOXO3a binds with the promoter of LC3 to induce autophagy and suppress renal tubular epithelial cell injury 146. Additionally, the SIRT3 regulated NRF2/HO-1 pathway may also contribute to inhibiting the formation of kidney stones 147. Furthermore, SIRT3 can inhibit renal fibrosis. Endothelial-to-mesenchymal transition (EndoMT) has emerged as an important contributor to renal fibrosis. Mice with SIRT3 loss more easily develop renal dysfunction with increased ROS production and EndoMT. Interestingly, SIRT3 can activate FOXO3a to inhibit this progress 148. SIRT3 can also inhibit renal fibrosis by deacetylation and activation of GSK3 β to inhibit the expression of fibrosis genes 133. Liraglutide can protect the kidney from diabetic nephropathy, while deletion of SIRT3 abrogated its kidney protection effect 31. Optic atrophy 1 (OPA1), a regulator of mitochondrial fusion, can be up-regulated by SIRT3, which subsequently enhances the fusion of renal mitochondria and improve the production of kidney energy 142. In short, SIRT3 can protect the kidney from metabolic disease.

Liver is the main metabolic organ of the human body. It governs the metabolism of glucose, protein, fat and carbohydrates. Also, it is also the largest detoxification organ in the human body. Abnormal liver metabolism will induce a series of hepatic metabolic diseases, of which fatty liver is the most common. SIRT3 is always down-regulated in patients with fatty liver 149-151. SIRT3 overexpression can restore liver function, inhibit inflammation and apoptosis, and alleviate liver fibrosis. SIRT3 activates ERK-CREB signaling to promote BNIP3 activity, thus inducing BNIP3 regulated mitotic resistance to nonalcoholic fatty liver disease 31. SIRT3-induced autophagy can also protect the liver from alcohol-induced injury 152. However, there are often two sides to complex issues. Another study demonstrated that, in contrast, SIRT3 could be a negative regulator of autophagy, which can lead to NAFLD induced by lipotoxicity 153. More interestingly, SIRT3 can repress hepatitis caused by infection with the Hepatitis B virus (HBV). In a recent study, SIRT3 was found to aggregate in the covalently closed circular DNA (cccDNA) of HBV and deacetylate Lys 9 of Histone H3, inhibiting HBV transcription and replication 154. As it does in other organs, SIRT3 can enhance ROS clearance to protect liver from oxidative damage by activating MnSOD2 and FOXO3a. Also SIRT3 protects hepatocytes by inhibiting mitochondrial damage and apoptosis through deacetylation of Ku70 155, 156. In addition, SIRT3 can alleviate liver fibrosis. The deacetylation and activation of GSK3β at Lys 15 by SIRT3 inhibit the expression of fibrosis genes in the liver 133. ROS promotes liver fibrosis through AKT-mTOR and ERK1/2 signaling, while SIRT3 resists liver fibrosis by eliminating harmful ROS. SIRT3 is definitely a liver protector by safeguarding metabolic stability.

**Figure 6:**
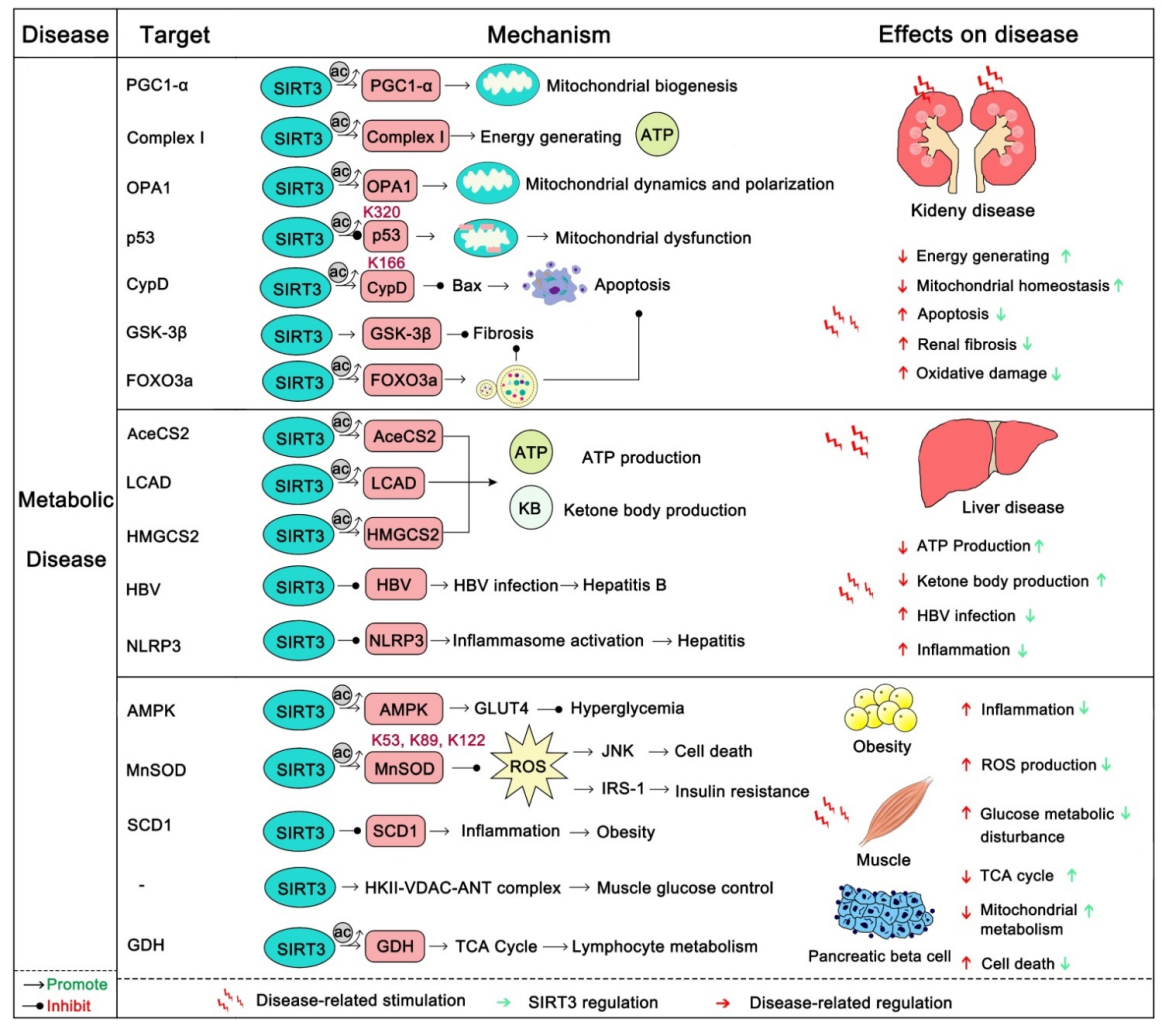
** SIRT3 in metabolic disease.** The body's energy metabolism, glycometabolism, fatty acid metabolism, and amino acid metabolism are generally imbalanced in metabolic diseases. SIRT3 can regulate a series of substrates to maintain the metabolic balance and stability of different organisms, and inhibit the occurrence and development of metabolic diseases. In addition, SIRT3 can inhibit the fibrosis of each organ and protect its normal function. It is worth noting that SIRT3 is also involved in the fight against viral infections and inflammatory responses. The pink protein represents the substrate for SIRT3. Gray circles represent acetylation modifications.

Obesity and diabetes are becoming ever more common around the world and are now trending with younger people. Although a recent study suggests that SIRT3 is dispensable in adipocyte metabolism and obesity-induced metabolic complications 157, SIRT3 in fact is important in obesity and diabetes. Of note, insulin resistance and vascular dysfunction are common in obese patients, which are exacerbated by SIRT3 deficiency 158, 159. SIRT3 can regulate endothelial cell glycolytic metabolism, and mice lacking the expression of SIRT3 will increase insulin resistance because of the dysfunction of glucose uptake and mitochondrial function 119, 160. In addition, SIRT3 can protect endothelial cells from mitochondrial ROS damage and increase NO release to benefit vasodilatation 159. Moreover, SIRT3-mediated SOD2 deacetylation also contributes to maintaining the escape of endothelial progenitor cells (EPCs) from dysfunction and injury as well as decreased vascular inflammation 161, 162. Maternal obesity is another key problem in human beings. The resulting high oxidative stress and meiotic defects of oocytes will damage reproductive health. Activation 7of SIRT3 can attenuate oxidative damage and improve the oocyte quality in obese woman 163. Of note, the deficiency of SIRT3 causes pancreatic beta cells to be more sensitive to cellular stress and oxidative damage, impairs their function and promotes the development of diabetes 164. Activation of SIRT3 can also regulate skeletal muscle metabolism and activate insulin signaling to improve diabetes. In this process, SIRT3 removes ROS to inhibit the activation of JNK and ISR-1 165. Interestingly, activation of pro-inflammatory macrophages is fateful in the pathogenesis of insulin resistance in diabetes. Loss of SIRT3 leads to the increasing expression of inflammatory cytokines and high risk of diabetes 166. Pyruvate dehydrogenase (PDH) is a key regulator in the TCA cycle and glucose oxidation. SIRT3 can deacetylate its E1α subunit to increase its enzymatic activity 167. SIRT3 also promotes HKII-VDAC-ANT complex formation to improve glucose control 168. As it does in other diseases, SIRT3 activates MnSOD2 and IDH2 to remove harmful ROS and activates FOXO3a to induce cell-protective autophagy to improve obesity and diabetes. Thus, SIRT3 protects human from metabolic diseases (Figure 6), and SIRT3 modulators will shine in the prevention and early treatment of metabolic diseases.

### SIRT3 in other diseases

In addition to several major diseases, SIRT3 offers protection from other common diseases and improves the quality of human life. SIRT3 can maintain bone metabolism by enhancing the AMPK-PGC-1β axis 169. SIRT3 can also protect microvasculature from LPS-induced damage. LPS upregulates Ang-2, leading to vascular leakage, while SIRT3 inhibits the expression of Ang-2 to maintain vascular integrity 170. Up-regulation of SIRT3 can attenuate endothelial cellular senescence to decrease the risk of atherosclerosis 171. In addition, activation of SIRT3 can suppress osteoarthritis by maintaining mitochondrial metabolism stability 172. Overall, SIRT3 is very important in maintaining human health and proper regulation of SIRT3 will be beneficial to human health.

## Targeting SIRT3 for potential therapies

As a result of the importance of SIRT3 in various diseases, several SIRT3 regulatory compounds have been discovered or designed synthetically. These compounds can deacetylate SIRT3 or regulate its expression level through different mechanisms. Based on their influence on SIRT3, these compounds were divided into two broad categories, SIRT3 activators (strictly speaking, “positive modulators”) and inhibitors. In this section we will discuss these compounds, the related mechanisms and potential therapeutic implications.

### Positive modulators of SIRT3

SIRT3 dysfunction is closely related to the occurrence and development of various diseases, and activation of SIRT3 appears to be an effective strategy for the treatment of many diseases. Unfortunately, to date SIRT3 agonist has ever been reported to date. Several positive modulators of SIRT3 were reported, which can stimulate SIRT3 by elevating SIRT3 expression. By upregulating SIRT3, they have displayed promising therapeutic effects in some diseases such as cardiac hypertrophy, acute kidney injury, and others (Table 3). Of note, most positive regulators of SIRT3 are derived from natural products. For instance, Honokiol is one of the most studied SIRT3 activators and is a natural lignan derived from the bark of Magnolia. Honokiol could increase SIRT3 expression and deacetylation activity, which have a favorable effect on heart disease 16, 137, renal disease 173, surgery/anesthesia-induced cognitive decline 174 and Vitiligo 175. For heart disease, Honokiol activation of SIRT3 further decreases the acetylation levels of MnSOD2 and OSCP, resulting in improved mitochondrial rate of oxygen consumption and inhibition of ROS synthesis. In addition, Honokiol suppresses cardiac hypertrophy and fibrosis via SIRT3-regulated AKT and ERK1/2 inhibition in mice with cardiac hypertrophy 16. Furthermore, in mice with doxorubicin-induced cardiomyopathy, Honokiol activated SIRT3 to promote mitochondrial fusion and inhibit apoptosis. More intriguingly, activation of SIRT3 by Honokiol can protect against doxorubicin-induced cardiotoxicity in tumor-xenograft mice without affecting the anti-tumor effect of doxorubicin. This makes it possible for Honokiol to contribute to adjuvant therapy of chemotherapy 137. In renal disease, Honokiol stimulates SIRT3 activity to block the NF-κB-TGF- β1/Smad regulated inflammation and fibrosis signaling in renal fibrosis mice model 173. Surprisingly, Honokiol can ameliorate surgery-induced or anesthesia-induced cognitive decline in mice via SIRT3 activation mediating ROS elimination and apoptosis inhibition 174. Interestingly, honokiol could improve vitiligo via melanocyte apoptosis inhibition through SIRT3-OPA1 axis activation 175. These studies suggest that Honokiol may be helpful in the treatment of various diseases, whether used alone or as an adjunct therapy. Silybin is another natural plant-derived SIRT3 activator. It is isolated from the seeds of blessed milkthistle (Silybum marianum) and is used as a hepatoprotectant in traditional Chinese medicine. It was reported that Silybin can improve kidney mitochondrial function via activating SIRT3 in a mouse model of cisplatin-induced acute kidney injury. Up-regulation of SIRT3 eliminates ROS and inhibits apoptosis to protect kidney cells from death. It is noteworthy that this protective effect can decrease cisplatin-induced renal toxicity and may contribute to clinical adjuvant treatment in cisplatin chemotherapy 176. Resveratrol, a famous SIRT1 activator (although resveratrol-mediated activation of sirtuins has been repeatedly shown to be an experimental artifact, it does have biological activity* in vivo*), can also increase the expression of SIRT3 to attenuate acute kidney injury 177. Fascinatingly, Dihydromyricetin has a similar chemical structure to Resveratrol and it can elevate the expression of SIRT3 through SIRT3 mediated cytoprotection and inflammatory resistance, thus treating osteoarthritis 172. Polydatin, a polyphenolic compound isolated from Polygonum cuspidatum, can initiate SIRT3-regulated mitochondrial autophagy to protect cardiomyocytes from myocardial infarction 178. Moreover, in a mouse model of sulfur mustard-induced hepatic injury, this natural product exerts its hepatoprotective effects via SIRT3 179. Pyrroloquinoline quinone, another natural product, can improve liver metabolic diseases through increasing the expression of SIRT3 180.

Importantly, a growing number of reports show that some “old drugs” have a capacity of activating SIRT3 with a clear action mechanism. Metformin is a known AMPK activator which applied as the first-line drug for type 2 diabetes. Metformin can improve atherosclerosis aroused by type 2 diabetes via up-regulating SIRT3 181. Hearing loss is a common age-related disease with cell degeneration. The Cl^-^ channel blocker, Adjudin, prevents gentamicin-induced hair cell loss via SIRT3 182. Interestingly, a hormone named Melatonin, which targets the melatonin receptor to play a role in sleep, now has been found to be a SIRT3 activator that plays a protective role in heart disease 183, liver injury 184 and atherosclerosis 185. In a mouse model of myocardial ischemia/reperfusion (MI/R) injury, melatonin also increases SIRT3 expression and activity to inhibit apoptosis of cardiomyocytes and maintain the stability of mitochondrial metabolism 183. In addition, in cadmium-induced hepatotoxicity *in vitro* and *in vivo* models, melatonin enhances the activity of SIRT3 to inhibit both ROS production and cadmium-triggered autophagic cell death 184. Additionally, in a mouse model of atherosclerosis, melatonin activates SIRT3-FOXO3a-Parkin regulated mitophagy to prevent inflammation and atherosclerotic progression 185.

Another type of SIRT3 activator is a substrate-dependent activator. 7-hydroxy-3-(4'-methoxyphenyl) coumarin (C12) is a compound that promotes the deacetylation of MnSODK68AcK (MnSOD acetylated at Lys 68) through SIRT3. C12 was identified based on the crystal structure of MnSODK68AcK. C12 binds to the MnSODK68AcK-SIRT3 complex and promotes the deacetylation and activation of MnSOD. The Kd value of C12 binds to SIRT3 is 3.9 μM and the 50% activation concentration is 75.78 μM towards MnSODK68AcK 15. Of note, among all these SIRT3 activators, C12 is closest to the real SIRT3 activator.

As a fascinating target for disease treatment, SIRT3 has always been a research hotspot. Although these positive modulators of SIRT3 have certain therapeutic potentials for disease, the design of targeted small-molecule activators of SIRT3 still faces significant challenges.

The design of SIRT3 activators, which control the SIRT3 catalytic activity level by some exact mechanism, lacks a theoretical and structural basis. At present, allosteric activators have attracted much attention in the design of targeted activators of SIRTs (such as SIRT1 and SIRT6). Further elucidation of the structure and biological function of SIRT3 will promote the development of small molecule activators targeting SIRT3.

### SIRT3 inhibitors

**Figure 7:**
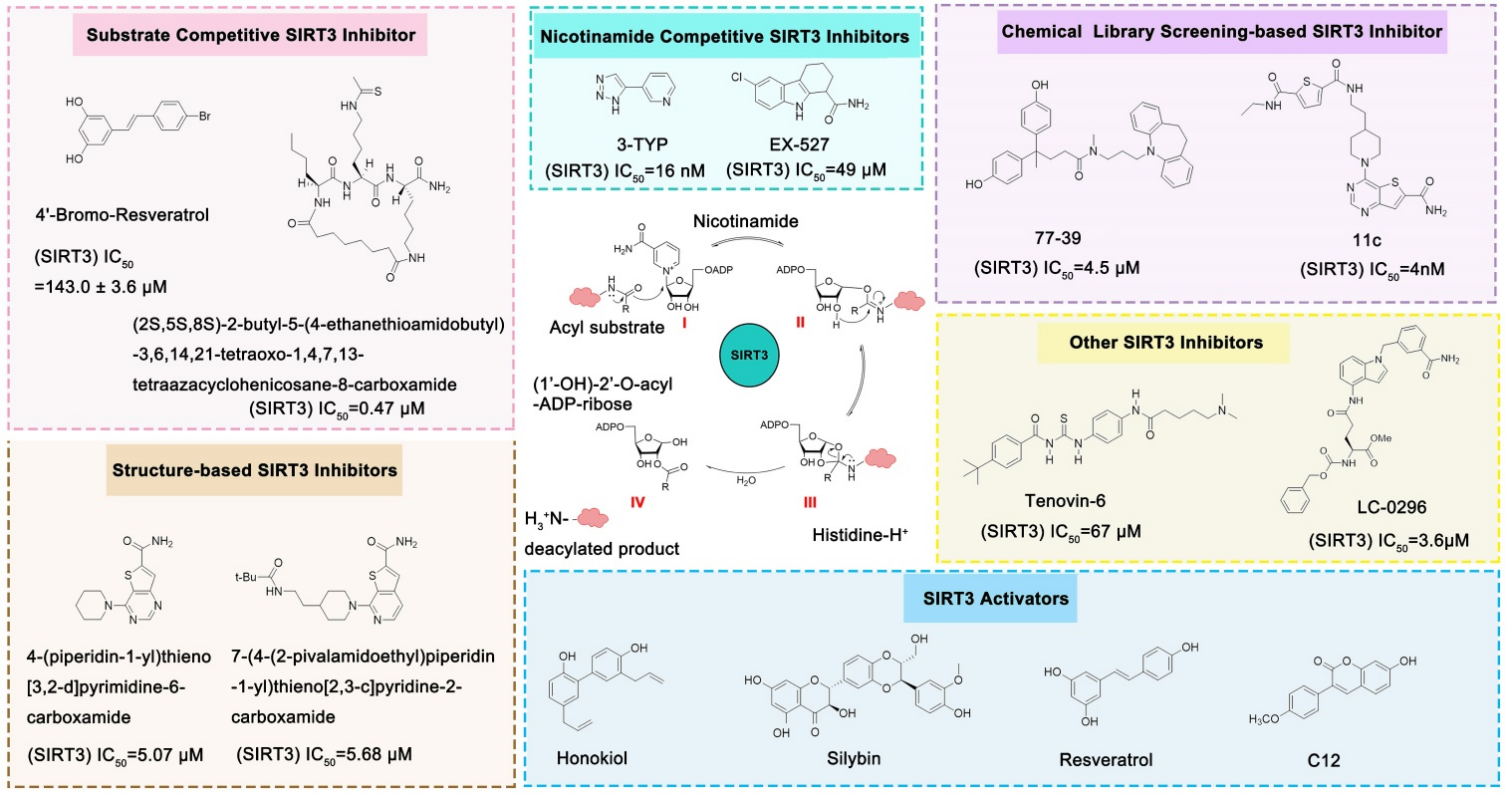
** The NAD^+^-dependent SIRT3 deacetylation reaction process, SIRT3 activators and SIRT3 inhibitors.** The NAD^+^-dependent SIRT3 deacetylation reaction process is roughly divided into four steps. I, The acetylated substrate and the NAD^+^ co-substrate binding to SIRT3. II, the acetyl group consequently transfer from substrate to ADP-ribose moiety of NAD^+^. III, Generation of bicyclic intermediates. IV, Produce the deacetylated protein. SIRT3 inhibitors are divided into five types. Substrate competitive SIRT3 inhibitors, Nicotinamide competitive SIRT3 inhibitors, chemical library screening-based SIRT3 inhibitors, structure-based SIRT3 inhibitors and other SIRT3 inhibitors. The chemical structures of representative SIRT3 inhibitors and activators are displayed in the figure.

Compared with the discovery of SIRT3 activators, the development of SIRT3 inhibitors has been much easier. The deacetylation process of SIRT3 (Figure 7) logically suggests a series of methods to inhibit this chemical reaction. In addition, as the crystal structure of the SIRT3 protein has been identified, another strategy for the discovery of SIRT3 inhibitors is structure-based design. Last but not least, chemical library screening is another common way to discover SIRT3 inhibitors 17. Of note, another type of SIRT3 inhibitor has been found fortuitously, which can inhibit SIRT3 expression or activity 186, 187. Through these strategies, a variety of SIRT3 inhibitors have been developed and they have shown good viability in a variety of diseases (Table 4), especially in cancer.

#### Catalytic Mechanism-based SIRT3 Inhibitors

The deacetylation of SIRT3 is a multi-step, continuous and complex process involving the coenzyme NAD^+^ and the acetylated substrate. Targeting the dynamic process of deacetylation is an effective strategy for the design of SIRT3 inhibitors. On one hand, structural analogues of endogenous acetylated substrates can effectively and competitively inhibit the deacetylation activity of SIRT3. On the other hand, NAD^+^ coenzyme competitive inhibitors can accelerate the reverse reaction of deacetylation to inhibit the progress of the deacetylate reaction. Notably, it is easy to achieve considerable satisfactory inhibition efficiency and selectivity with the catalytic mechanism-based inhibitors. This is the fascinating characteristic of this type of SIRT3 inhibitor and, of course, why it is one of the most studied.

#### Substrate Competitive SIRT3 Inhibitors

**Figure 8:**
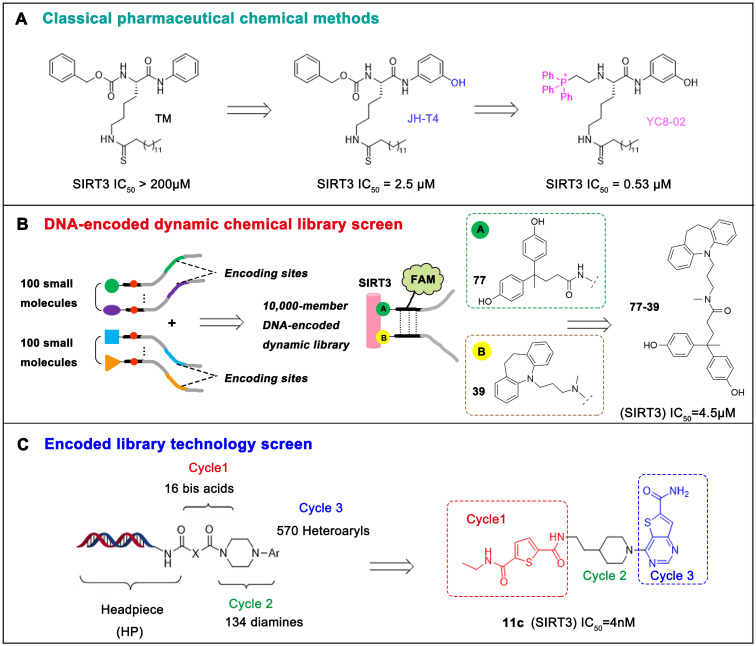
** Potential therapeutic strategies of representative SIRT3 inhibitors.** (**A**) SIRT3 inhibitor discovered by classical pharmaceutical chemical method. (**B**) SIRT3 inhibitor discovered by DNA-encoded dynamic chemical library screen strategy. (**C**) SIRT3 inhibitor discovered by encoded library technology screen method.

The most common and effective inhibitor design strategies use substrate competitive inhibitors. 4'-bromo-resveratrol is an ACS2 peptide substrate competitive inhibitor discovered in 2013 188. Recently, 4'-bromo-resveratrol was found to inhibit melanoma progression via SIRT3 mediated mitochondrial metabolic reprogramming. In this process, 4'-bromo-resveratrol was also confirmed to induce apoptosis and G0/G1 cell cycle arrest 189. Simon et al. discovered a peptide substrate competitive SIRT1/2 inhibitor, cambinol, which has a potential effect on cancer treatment. After that, they designed a series of cambinol analogues and discovered the SIRT3 selective inhibitor 4- [(2-Hydroxy-6-phenylnaphthalen-1-yl) methyl]-5-(4-methylphenyl) -2, 3-dihydro-1H-pyrazol-3-one (SIRT3 IC_50_ = 6 μM) that has good anti-cancer potential 190. Analogs of N^Ɛ^-acyl-lysine are important substrate competitive SIRT3 inhibitors. Peptide-based SIRT3 inhibitors are one of the analogs of N^Ɛ^-acyl-lysine with excellent proteolytic stability and cell permeability. Chen et al. designed a series of peptides containing N^Ɛ^-thioacetyl-lysine that exhibit SIRT1/2/3 inhibition effects. Their effects vary from 0.47 μM to 10.8 μM and the most potent one can efficiently inhibit SIRTs activity in HCT116 human colon cancer cells. Unfortunately, despite its acceptable SIRT3 inhibitory activity, this one is not a selective SIRT3 inhibitor (SIRT3 IC_50_ = 0.22 μM; SIRT3 IC_50_ = 0.24 μM; SIRT3 IC_50_ = 0.47 μM) 191. This also suggests that if we want to develop specific substrate-competitive SIRT3 inhibitors, we may need to select SIRT3-specific substrate action sites for targeted design. Zheng et al. also designed a variety of potent SIRT3 tripeptidic inhibitors containing N^Ɛ^-thioacetyl-lysine. However, their selectivity was generally not strong 192. SIRT3-selective inhibitors can be obtained from other SIRT inhibitors by classical medicinal chemistry methods 108. One case in point is the thioacyl lysine compound TM reported by Hui Jing et al. It was identified as targeting SIRT2 with little SIRT3 inhibition. 3-hydroxy substitution (generating JH-T4) remarkably enhances SIRT3 inhibition with an IC_50_ of 2.5 μM. Considering the fact that SIRT3 is abundant in mitochondria, researchers modified JH-T4 by replacing the benzyl carbamoyl group with a triphenylphosphonium (TPP) mitochondrial targeting moiety to obtain the compound YC8-02, which achieved superior penetration into mitochondria. Accordingly, the SIRT3 IC_50_ was further increased to 0.53 μM. YC8-02 not only exhibits strong SIRT3 inhibition effects, but also inhibits lymphomagenesis by selectively inhibiting SIRT3 (Figure 8A) 108.

#### Nicotinamide Competitive SIRT3 Inhibitors

Nicotinamide is an endogenous inhibitor of SIRTs, but the one major drawback is that it has no selectivity for SIRTs. However, it is undeniable that nicotinamide analogues are an important component of SIRT3 inhibitors. 3-TYP is essentially a nicotinamide analogue which is a widely used tool in medicine with high selective SIRT3 inhibition (SIRT3 IC_50_=16 nM) 193. EX-527 is a selective SIRTs inhibitor that functions by occupying the nicotinamide site and neighboring pocket contacting NAD^+^. EX-527 always been defied as a SIRT1 inhibitor, but it can also inhibit SIRT3 with weak activity (SIRT3 IC_50_=49 μM) 194. Interestingly, the discovery of EX-527 revealed a new SIRT inhibition mechanism, namely, the formation of trimer Sirtuin complex with a NAD^+^-derived coproduct.

#### Structure-based SIRT3 Inhibitors

Docking and binding free energy calculations are methods to discover novel SIRT3 inhibitors based on its reported structure. In this way, Berin et al. discovered a fair number of compounds that exhibit fine SIRT3 inhibition effects. Unfortunately, their biological activity has not been detected, so their application may require more work to support 195.

#### Chemical Library Screening-based SIRT3 Inhibitors

Chemical library screening is an effective way to discover novel SIRT3 inhibitors. Recently, the novel SIRT3 inhibitor **77-39** (SIRT3 IC_50_ = 4.5 μM) was discovered through the DNA-encoded Dynamic Chemical Library (Figure 8B). **77-39** also exhibits excellent cellular SIRT3 inhibition activity, which increases the global mitochondrial acetylation level of HeLa cells. Moreover, **77-39** showed only little cytotoxicity 194. Encoded Library Technology screening is another efficient strategy to identify novel SIRT3 inhibitors. Through this strategy, the potent SIRT3 inhibitor **11c** ((N2-(2-(1-(6-carbamoylthieno [3, 2-d] pyrimidin-4-yl) piperidin-4-yl) ethyl)-N5-ethylthiophene-2, 5-dicarboxamide)) (SIRT3 IC_50_ = 4.0 nM) was discovered (Figure 8C) 197.

#### Other SIRT3 Inhibitors

Tenovin-6 is a p53 activator with biological activity that later was also found to be a SIRT3 inhibitor (SIRT3 IC_50_ = 67 μM) with anti-tumor activity. The inhibitory effect on SIRT3 was unclear, but it has been proven to act as a non-competitive inhibitor 186. LC-0296 is a synthetic SIRT3 inhibitor with good inhibitory effect (SIRT3 IC_50_ = 3.6 μM) and its mechanism is also unclear. Judging by its structure, LC-0296 might be a NAD^+^ competitive inhibitor. It has good activity against squamous cell carcinoma of the head and neck through inhibiting proliferation and promoting apoptosis 198. Trimethylamine-N-oxide (TMAO) is a choline metabolite that can promote vascular inflammation through SIRT3 inhibition-induced ROS-NLRP3 activation 199. Albendazole is an anthelmintic drug with microtubule-targeting ability. Recently, albendazole was found to induce SIRT3 degradation to inhibit leukemia cell survival 187. 2-methoxyestradiol (2-ME) is an anti-cancer drug, which has been found to bind to the typical and allosteric inhibitor binding sites on SIRT3 to inhibit its activity. By inhibiting SIRT3, 2-ME can disturb normal mitochondrial functions and kill osteosarcoma cells 200.

Although the design and discovery of SIRT3 inhibitors are easier than activators, the development of proprietary and highly efficient SIRT3 inhibitors remains a challenge. The application of SIRT3 inhibitors is something that requires more attention. Given the cytoprotective role of SIRT3 in multiple organs, inhibition of SIRT3 in certain cancers should be carefully evaluated and discussed. Therefore, how to develop a highly efficient SIRT3 inhibitor without organ toxicity is widely expected. In addition, the introduction of new drug delivery methods, such as target organ delivery, may open a new door for the application of SIRT3 inhibitors.

## Conclusions

SIRT3, the major deacetylase in mitochondria, has been well-established to be involved in all aspects of mitochondrial metabolism as well as the synthesis and movement of mitochondria. Mitochondria play a very important role in cells, and their disorders induce a variety of diseases. The dysregulation of SIRT3 has been confirmed in many mitochondria-related diseases.

An accumulation of current evidence has gradually revealed the mysteries of the inherent biological functions of SIRT3 and medicinal applications in human diseases. SIRT3 has been attracting extensive attention from its original role as the longevity gene to being a potential “superstar” target in many diseases. Several studies have demonstrated that the improved role of SIRT3 in many diseases is mainly due to its scavenging effect on ROS. Excessive accumulation of ROS is often an important factor in the development of age-related diseases, heart disease, cancer and metabolic diseases. SIRT3 activates its substrates such as MnSOD2, IDH2 and PHD by deacetylation and then removes excess ROS. On the other hand, SIRT3 regulates TCA, OXPHOS, fatty acid and amino acid metabolism to maintain mitochondria homeostasis in normal or slightly damaged mitochondria to influence cell fitness and survival. However, when the mitochondria are too damaged to be repaired, SIRT3 can deacetylate and activate FOXO3a to induce mitophagy, thus eliminating and circulating mitochondria. Interestingly, regarding to its inherent complexity, SIRT3 has been demonstrated to play the Janus role in cancer, indicating that SIRT3 activators or inhibitors would be utilized as anti-tumor agents in specific types of cancers, respectively. For instance, it cannot be ignored that the application of SIRT3 inhibitors in some OXPHOS-addiction cancers seems very promising, but in regarding to the protective effects of SIRT3 on various human organs, inhibition of SIRT3 might partially bring multiple organ toxicities. On the other hand, the excessive pursuit of SIRT3 activation might also lead to unpredictable carcinogenesis under some circumstances. Thus, considering either up-regulation or down-regulation expression of SIRT3 seems to be so limited for the discovery of new small-molecule inhibitor or activator in the treatment of different types of diseases. Maybe, the combination of SIRT3 activator/inhibitor and the other drug in a new delivery system targeting specific human organ would be an alternative potential therapeutic strategy.

Hitherto, no specific SIRT3 activators or potent inhibitors have been successfully discovered and thus being utilized for potential therapeutics. But still, several small-molecule compounds with therapeutic potential have been reported. For example, the most famous SIRT3 activator, Honokiol, exhibits some therapeutic effects in heart disease, inflammation-related diseases, cancer and metabolic diseases. However, its beneficial effects are not entirely attributed to the activation of SIRT3. The structural characteristics of its polyphenols determine its outstanding antioxidant capacity, which is also the key to its therapeutic activity. Similarly, although the highly selective SIRT3 inhibitor 3-TYP has good properties, it is only used as a probe with almost no therapeutic applications.

Therefore, how to develop an effective SIRT3 activator or inhibitor for therapeutic purposes remains to be an enigma. In this regard, scientific drug design strategy is particularly important. For the design of SIRT3 activators, there is still a lack of effective and scientific design methods at present. Because the mechanism of the interaction between small molecule agonists and SIRT3 is still a black box, there is no basis for structure-based drug design. At present, allosteric agonists are one of the most convincing methods for the design of SIRT3 agonists. Recently, artificial intelligence has been applied in the field of drug design, which has opened a new era of revolutionary drug design. However, much effort still needs to be made to elucidate the molecular mechanism of SIRT3 allosteric activation.

As a plan for SIRT3 inhibitor design, structure-based drug design is a mature and effective strategy, including receptor- and ligand-based technology. Among them, basing this on the binding characteristics of the substrate and SIRT3 complex is a better choice for selective SIRT3 inhibitors. Co-enzyme structure-based design is more likely to lose selectivity, resulting in serious side effects. Additionally, PROTAC technology is a new strategy to design potent targeted inhibitors. Taken together, SIRT3, from a mitochondrial metabolic regulator to a promising therapeutic target, would shed new light on exploiting more candidate drugs for fighting human diseases in the near future.

## Figures and Tables

**Table 1:** Classification, function and characteristics of mammal sirtuins

Sirtuin	Class	Enzymatic activity	Subcellular localization	Function	Disease	Reference
SIRT1	I	Deacetylase	Cytoplasm & Nuclear	a. Metabolism regulation b. Regulation of chromatin and transcription c. DNA repair d. Inflammation suppression	a. Aging b. Neurodegeneration c. Metabolic disease d. Cardiovascular disease e. Cancer	1, 7
SIRT2	I	Deacetylase	Cytoplasmic & Nuclear	a. Cell differentiation b. Metabolism regulation c. Cell cycle regulation d. Microtubule dynamics e. Inflammation regulation	a. Cancer b Neurodegeneration	1, 7, 8
SIRT3	I	Deacetylase	Mitochondria & Nuclear	a. Metabolism regulation b. Inflammation suppression c. Inhibition of oxidative stress d. Apoptosis regulation e. Autophagy regulation	a. Aging b. Neurodegeneration c. Metabolic disease d. Cardiovascular disease e. Cancer	1, 7
SIRT4	II	a. ADP-ribosyltransferase b. Lipoamidase c. Deacetylase	Mitochondria	Metabolism regulation	a. Metabolic disease b. Cancer c. Neurodegeneration	1, 7
SIRT5	III	a. Deacetylase b. Desuccinylase c. Demalonylase d. Deglutarylase	Mitochondria	a. Metabolism regulation b. Immune response modulation	a. Metabolic disease b. Cancer	1, 7, 9
SIRT6	IV	a. ADP-ribosyltransferase b. Deacylase c. Deacetylase	Nuclear	a. Chromatin and DNA repair b. Metabolism regulation	a. Aging b. Cancer c. Metabolic disease d. Cardiovascular disease	1, 4, 7, 10
SIRT7	IV	Deacetylase	Nuclear	a. Transcription regulation b. Chromatin remodeling c. Metabolism regulation	a. Cancer b. Metabolic disease c. Cardiovascular disease	1, 7, 11

**Table 2:** Endogenous direct regulators of SIRT3

Name	Classification	Regulatory Mechanism	References
NAD^+^	cofactor	Promotes the deacetylation process of SIRT3	20
Nicotinamide	Deacetylation product	Nicotinamide inhibits SIRT3 through rebinding of the reaction product to the enzyme accelerates the reverse reaction	17, 21
Caloric Restriction	-	Increases SIRT3 expression and activity	20
MPP	Peptidase	Proteolytic processing of FLSIRT3 to active SIRT3	22
SENP1	SUMOspecific protease	SENP1 can de-SUMOylates and activates SIRT3	23
4-Hydroxynonenal	Endogenous product	4-Hydroxynonenal inhibits SIRT3 activity by occupy its zinc-binding residue Cys(280).	24
NF-κB	Transcription factor	NF-κB binds to the SIRT3 promoter to enhance its expression	25
PGC-1α	Transcriptional coactivator	PGC-1α bounds to the SIRT3 promoter as its transcription factor to regulate SIRT3 expression	26, 27
SNAI1	Transcriptional repressor	SNAI1 inhibits SIRT3 promoter activity	28
ZEB1	Transcriptional repressor	ZEB1 inhibits SIRT3 promoter activity.	29
miR-195	MicroRNA	miR-195 down-regulates SIRT3 expression through direct 3'-untranslated region targeting	31
miR-421	MicroRNA	miR-421 targets the 3'UTR of SIRT3 and decreases SIRT3 protein level	32
miR-494-3p	MicroRNA	miR-494-3p targets the 3'UTR of SIRT3 and inhibits SIRT3 expression at mRNA and protein levels	33
miR -708-5p	MicroRNA	MiRNA-708-5p targets the 3'UTR of SIRT3 and decreases SIRT3 protein level	34
miR-31	MicroRNA	miR-31 directly targets SIRT3 to repress its expression	35
miR-145	MicroRNA	miR-31 directly targets SIRT3 to reduce its expression	36
miR-298	MicroRNA	miR-298 directly targets SIRT3 to inhibit its expression	37
miR-210	MicroRNA	miR-210 targets and represses ISCU to change the NAD+/NADH ratio thus indirectly negative regulate SIRT3	38
TUG1	Long non-coding RNA	TUG1 negatively regulates the expression of miR-145 thus indirectly positively regulate SIRT3	36
DYNLRB2-2	Long non-coding RNA	DYNLRB2-2 suppresses the mRNA expression of miR-298 thus indirectly activate SIRT3	37
Profilin-1	Protein	Profilin-1 interacts with SIRT3 and promotes its expression	41
β-catenin	Protein	β-catenin suppresses SIRT3 promotor activity to negative regulate its expression	40

**Table 3:** Positive modulators of SIRT3

Compound	Chemical Structure	Target/Pathways	Disease/cell	Biological Activity	Reference
Honokiol	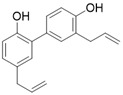	Honokiol increases SIRT3 expression and activity	a. Cardiac Hypertrophy b. Renal disease c. Surgery/anesthesia-induced cognitive decline d. Vitiligo	/	16, 137, 173-175
Silybin	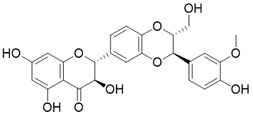	Silybin Increases SIRT3 expression	Acute Kidney Injury	/	176
Resveratrol	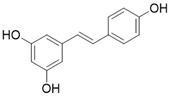	Resveratrol increases SIRT3 expression	Acute Kidney Injury	/	177
Polydatin	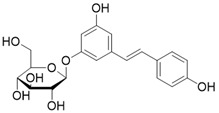	Polydatin increases SIRT3 activity	a. Myocardial infarction b. Ulfur mustard-induced hepatic injury	/	178, 179
Dihydromyricetin	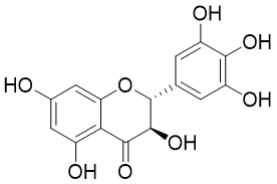	Dihydromyricetin increases the expression and activity of SIRT3 via activation of PGC-1α	Osteoarthritis	/	172
Pyrroloquinoline quinone	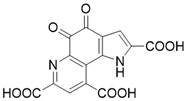	Pyrroloquinoline quinone increases the expression and activity of SIRT3	Liver metabolic diseases	/	180
Metformin	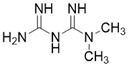	Metformin increases SIRT3 expression	Atherosclerosis	/	181
Adjudin	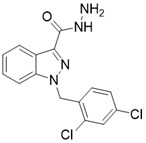	Adjudin increase the expression of SIRT3	Hearing loss	/	182
Melatonin	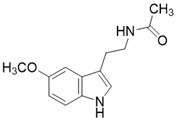	Melatonin activates SIRT3 signaling pathway	a. myocardial ischemia reperfusion injury b. liver injury c. atherosclerosis	/	183-185
7-hydroxy-3-(4'-methoxyphenyl) coumarin (C12)	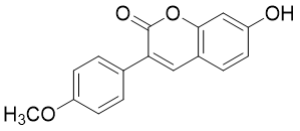	C12 binds to the MnSODK68AcK-SIRT3 complex and promotes the deacetylation and activation of MnSOD	unclear	(SIRT3)Kd=3.9μM; IC_50_(MnSODK68AcK)= 75.78 μM	15

**Table 4:** SIRT3 Inhibitors

Compound	Chemical Structure	Type	Disease/cell	Biological Activity	Reference
4'-Bromo-Resveratrol	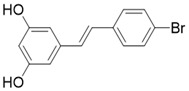	Substrate competitive SIRT3 inhibitor	Melanoma	SIRT3 IC_50_=143.0 ± 3.6 μM	188, 189
/(4- [(2-Hydroxy-6-phenylnaphthalen-1-yl) methyl]-5- (4-methylphenyl) -2,3-dihydro-1H-pyrazol-3-one)	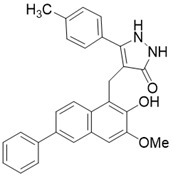	Substrate competitive SIRT3 inhibitor	Cancer	SIRT3 IC_50_=6 μM	190
(2S,5S,8S)-5-(4-ethanethioamidobutyl)-2-(naphthalen-2-ylmethyl)-3,6,13,20-tetraoxo-1,4,7,12-tetraazacycloicosane-8-carboxamide	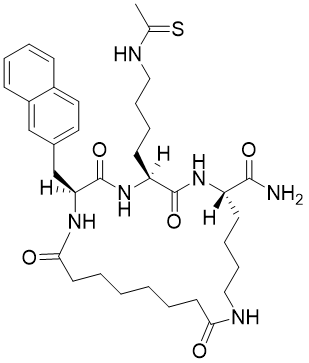	Substrate competitive SIRT3 inhibitor	Cancer	SIRT3 IC_50_=1.94 μM	190
(3S,6S,9S)-9-butyl-6-(4-ethanethioamidobutyl)-5,8,11,18-tetraoxo-1,4,7,10-tetraazacyclooctadecane-3-carboxamide	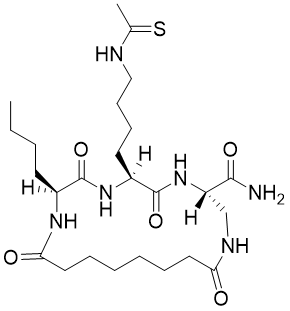	Substrate competitive SIRT3 inhibitor	Cancer	SIRT3 IC_50_=1.06 μM	191
(2S,5S,8S)-2-butyl-5-(4-ethanethioamidobutyl)-3,6,12,19-tetraoxo-1,4,7,11-tetraazacyclononadecane-8-carboxamide	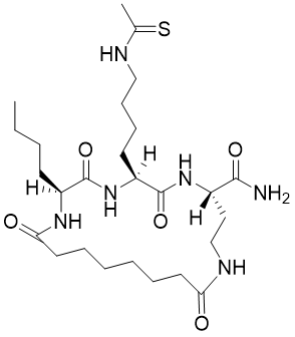	Substrate competitive SIRT3 inhibitor	Cancer	SIRT3 IC_50_=1.48 μM	191
(2S,5S,8S)-2-butyl-5-(4-ethanethioamidobutyl)-3,6,13,20-tetraoxo-1,4,7,12-tetraazacycloicosane-8-carboxamide	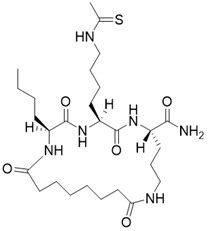	Substrate competitive SIRT3 inhibitor	Cancer	SIRT3 IC_50_=1.82 μM	191
(2S,5S,8S)-2-butyl-5-(4-ethanethioamidobutyl)-3,6,14,21-tetraoxo-1,4,7,13-tetraazacyclohenicosane-8-carboxamide	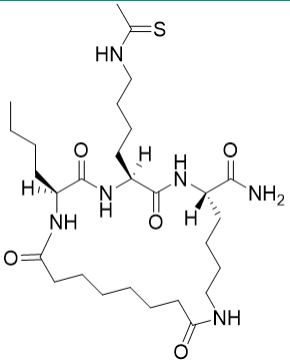	Substrate competitive SIRT3 inhibitor	Cancer	SIRT3 IC_50_=0.47 μM	191
(S)-2-((S)-4-([1,1'-biphenyl]-4-yl)-2-acetamidobutanamido)-N-((S)-6-acetamido-1-amino-1-oxohexan-2-yl)-6-ethanethioamidohexanamide	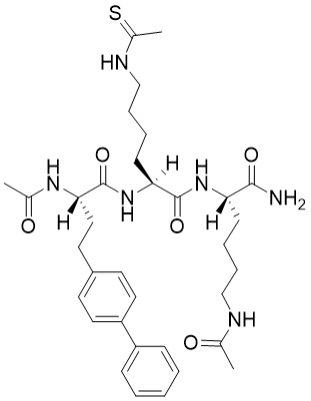	Substrate competitive SIRT3 inhibitor	/	SIRT3 IC_50_= 0.48 μM	192
N,N'-((S)-6-(((S)-1-(((S)-4-acetamido-1-amino-1-oxobutan-2-yl)amino)-6-ethanethioamido-1-oxohexan-2-yl)amino)-6-oxohexane-1,5-diyl)diacetamide	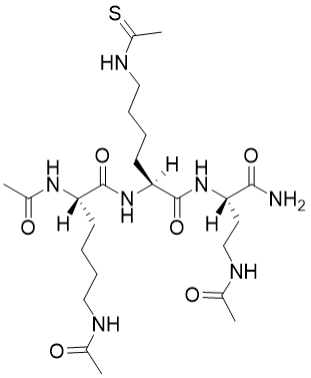	Substrate competitive SIRT3 inhibitor	/	SIRT3 IC_50_= 0.36 μM	192
N,N'-((S)-6-(((S)-1-(((S)-1-amino-1-oxohexan-2-yl)amino)-6-ethanethioamido-1-oxohexan-2-yl)amino)-6-oxohexane-1,5-diyl)diacetamide	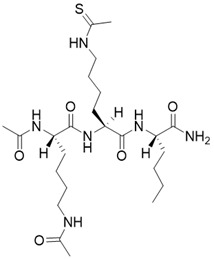	Substrate competitive SIRT3 inhibitor	/	SIRT3 IC_50_=0.48 μM	192
N,N'-((S)-6-(((S)-1-(((S)-1-amino-4-(naphthalen-2-yl)-1-oxobutan-2-yl)amino)-6-ethanethioamido-1-oxohexan-2-yl)amino)-6-oxohexane-1,5-diyl)diacetamide	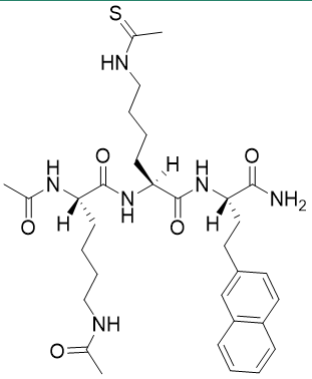	Substrate competitive SIRT3 inhibitor		SIRT3 IC_50_=2.1 μM	192
YC8-02	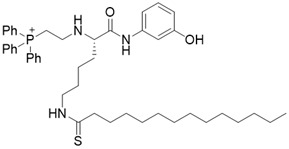	Substrate competitive SIRT3 inhibitor	Lymphoma	SIRT3 IC_50_=0.53 μM	108
JH-T4	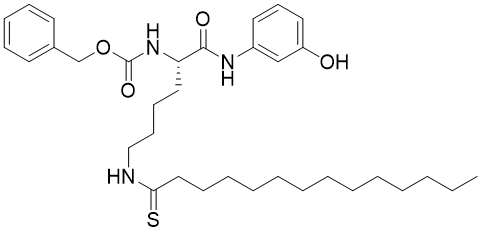	Substrate competitive SIRT3 inhibitor	Lymphoma	SIRT3 IC_50_=2.5 μM	108
3-TYP	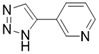	Nicotinamide competitive SIRT3 inhibitors	Tool medicine	SIRT3 IC_50_=16 nM	193
EX-527	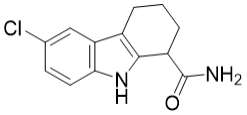	Nicotinamide competitive SIRT3 inhibitors	Cancer	SIRT3 IC_50_=49 μM	194
4-(4-(acetamidomethyl)piperidin-1-yl)thieno[3,2-d]pyrimidine-6-carboxamide	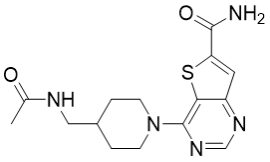	Structure-based SIRT3 inhibitors	/	SIRT3 IC_50_=5.36 μM	195
4-(4-(2-pivalamidoethyl)piperidin-1-yl)furo[3,2-d]pyrimidine-6-carboxamide	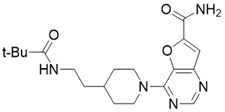	Structure-based SIRT3 inhibitors	/	SIRT3 IC_50_=5.89 μM	195
7-(4-(2-pivalamidoethyl)piperidin-1-yl)thieno[2,3-c]pyridine-2-carboxamide	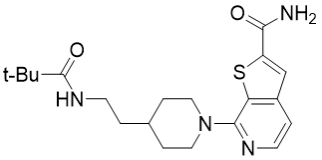	Structure-based SIRT3 inhibitors	/	SIRT3 IC_50_=5.68 μM	195
4-(piperidin-1-yl)thieno[3,2-d]pyrimidine-6-carboxamide	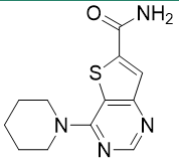	Structure-based SIRT3 inhibitors	/	SIRT3 IC_50_=5.07 μM	195
77-39	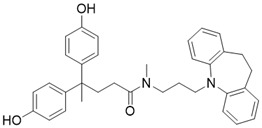	Chemical library screening-based SIRT3 inhibitor	/	SIRT3 IC_50_=4.5 μM; Kd =2.14 µM	196
11c (N2-(2-(1-(6-carbamoylthieno[3,2-d]pyrimidin-4-yl)piperidin-4-yl)ethyl)-N5-ethylthiophene-2,5-dicarboxamide)	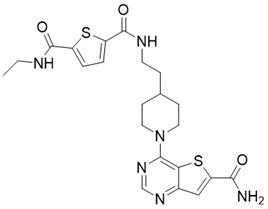	Chemical library screening-based SIRT3 inhibitors	/	SIRT3 IC_50_=4nM	197
Tenovin-6	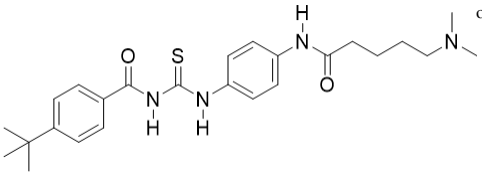	other	Cancer	SIRT3 IC_50_=67 μM	186
LC-0296	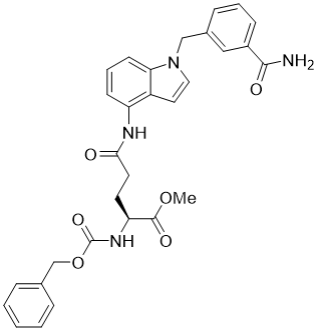	other	Head and Neck Cancer	SIRT3 IC_50_=3.6μM	198
Trimethylamine-N-oxide (TMAO)	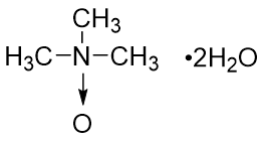	other	Vascular Inflammation	/	199
Albendazole	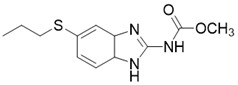	other	leukemia U937 and HL60 cells	/	187
2-methoxyestradiol	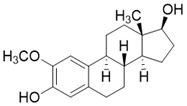	binding to both the canonical and allosteric inhibitor binding sites	Osteosarcoma Cancer	/	200

## Abbreviations

SIRT3: Sirtuin 3
TCA: tricarboxylic acid
UPR: unfolded protein response
OXPHOS: oxidative phosphorylation
CR: Caloric Restriction
IDH2: isocitrate dehydrogenase 2
FLSIRT3: full length SIRT3
MPP: Matrix processing peptidase
4-HNE: 4-Hydroxynonenal
PPARγ: Peroxisome proliferator-activated receptor γ
PGC-1α: PPARγ coactivator 1
miRNA: MicroRNA
ISCU: iron-sulfur cluster assembly protein
LncRNAs: Long non-coding RNA
AD: Alzheimer's disease
PD: Parkinson's disease
HD: Huntington's disease
AML: Amyotrophic lateral sclerosis
MnSOD2: manganous superoxide dismutase 2
ROS: reactive oxygen species
mPTP: mitochondrial permeability transition pore
NMNAT2: Nicotinamide mononucleotide adenylyltransferase 2
HSP90: heat shock protein 90
HIF1α: hypoxia-inducible factor-1a
PHD: prolyl hydroxylase
PDC: pyruvate dehydrogenase complex
Skp2: the F-box protein S-phase kinase associated protein 2
GSK-3β: glycogen synthase kinase-3β
ECHS1: Enoyl-CoA hydratase-1
GOT: Glutamate oxaloacetate transaminases
OGG1: 8-oxoguanine-DNA glycosylase 1
SHMT2: serine hydroxymethyltransferase 2
PYCR1: Pyrroline-5-carboxylate reductase 1
OPA1: Optic atrophy 1
OSCP: oligomycin-sensitivity conferring protein
CypD: cyclophilin D
LCAD: long-chain acyl CoA dehydrogenase
NMNAT3: Nicotinamide mononucleotide adenylyltransferase 3
STAT3: signal transducer and activator of transcription 3
AKI: acute kidney injury
EndoMT: Endothelial-to-mesenchymal transition
NAFLD: nonalcoholic fatty liver disease
HBV: Hepatitis B virus
cccDNA: covalently closed circular DNA
PDH: Pyruvate dehydrogenase
ZEB1: Zinc finger E-box-binding homeobox 1
GOT2: glutamate oxaloacetate transaminases 2
CLL: chronic lymphocytic leukemia
HCC: hepatocellular carcinoma
DLBCLs: diffuse large B cell lymphomas
EMT: epithelial-mesenchymal transition
TNBC: triple negative breast cancer
ACC1: acetyl-coA carboxylase 1
GLYAT: Glycine N-acyltransferase
HMGCL: hydroxymethylglutaryl-CoA lyase
EPCs: endothelial progenitor cells
